# Structural transitions in TCTP tumor protein upon binding to the anti-apoptotic protein family member Mcl-1

**DOI:** 10.1016/j.jbc.2023.104830

**Published:** 2023-05-16

**Authors:** Florian Malard, Christina Sizun, Aurélien Thureau, Ludovic Carlier, Ewen Lescop

**Affiliations:** 1 Institut de Chimie des Substances Naturelles, CNRS UPR 2301, Université Paris-Saclay, LabEx LERMIT, Gif-sur-Yvette, France; 2 Synchrotron SOLEIL, Saint Aubin, France; 3 Laboratoire Des Biomolécules, LBM, Sorbonne Université, Ecole Normale Supérieure, PSL University, CNRS, Paris, France

**Keywords:** Translationally Controlled Tumour Protein (TCTP), Mcl-1, apoptosis, protein-protein interaction, structural plasticity, cancer, NMR, SAXS

## Abstract

Translationally Controlled Tumor Protein (TCTP) serves as a pro-survival factor in tumor cells, inhibiting the mitochondrial apoptosis pathway by enhancing the function of anti-apoptotic Bcl-2 family members Mcl-1 and Bcl-xL. TCTP specifically binds to Bcl-xL, preventing Bax-dependent Bcl-xL-induced cytochrome c release, and it reduces Mcl-1 turnover by inhibiting its ubiquitination, thereby decreasing Mcl-1-mediated apoptosis. TCTP harbors a BH3-like motif that forms a β-strand buried in the globular domain of the protein. In contrast, the crystal structure of the TCTP BH3-like peptide in complex with the Bcl-2 family member Bcl-xL reveals an α-helical conformation for the BH3-like motif, suggesting significant structural changes upon complex formation. Employing biochemical and biophysical methods, including limited proteolysis, circular dichroism, NMR, and SAXS, we describe the TCTP complex with the Bcl-2 homolog Mcl-1. Our findings demonstrate that full-length TCTP binds to the BH3 binding groove of Mcl-1 *via* its BH3-like motif, experiencing conformational exchange at the interface on a micro- to milli-second timescale. Concurrently, the TCTP globular domain becomes destabilized, transitioning into a molten-globule state. Furthermore, we establish that the non-canonical residue D16 within the TCTP BH3-like motif reduces stability while enhancing the dynamics of the intermolecular interface. In conclusion, we detail the structural plasticity of TCTP and discuss its implications for partner interactions and future anticancer drug design strategies aimed at targeting TCTP complexes.

Translationally Controlled Tumor Protein (TCTP) is a small (20 kDa) single-domain globular protein widely conserved across phyla. TCTP, also called fortilin or Histamine Release Factor, interacts with many partners (1) and is consistently involved in numerous biological processes including tumorigenesis and tumor reversion (2, 3, 4, 5, 6). TCTP acts as an apoptosis inhibitor and promotes the proliferation of malignant cells. The pro-survival properties of TCTP in cancer cells are supported by numerous evidences such as protection from cell death induced by serum deprivation (7) or cytotoxic drugs (8, 9). Notably, TCTP is involved in a negative regulatory loop with the major tumor suppressor p53 (10, 11, 12, 13). TCTP pro-survival properties were also described in the context of interactions with the stress sensor IRE1α (14), the growth factor-beta stimulated clone-22 (TSC) (15) or by inhibition of the Ca^2+^-dependent apoptotic pathways (16).

Importantly, TCTP potentiates central inhibitors of the mitochondrial apoptosis pathway (17), namely, Bcl-xL (18, 19) and Mcl-1 (20, 21, 22). Bcl-xL and Mcl-1 are anti-apoptotic members of the Bcl-2 family proteins and they sequestrate pro-apoptotic factors such as Bax or Bak, respectively, thus inhibiting Mitochondrial Outer Membrane Permeabilization (MOMP) (23). In mitochondrial-based assays, the molecular interaction between TCTP and Bcl-xL inhibits Bax-dependent MOMP and cytochrome c release. On the other hand, TCTP also exerts its anti-apoptotic role by increasing Mcl-1 stability (20, 22). Indeed, TCTP inhibits the proteasome-mediated degradation of Mcl-1 by reducing its ubiquitinylation *via* a yet unknown mechanism (20). In the context of murine macrophage cell line infection by intramacrophage parasite *Leishmania donovani*, TCTP was found to be strongly associated with Mcl-1, which prevents ubiquitination-mediated degradation of Mcl-1 and inhibits apoptosis (22). It is well described that Mcl-1 is a target of the E3 ubiquitin-ligase Mule and that the interaction can be regulated by Bcl-2 family proteins that contain a BH3 motif (24). Hence, TCTP has been proposed to prevent Mule from ubiquinating Mcl-1 to increase the cellular stability of Mcl-1 (22). Mule also contains a BH3 motif that binds to the BH3-binding groove of Mcl-1 (see PDB code: 5C6H) which explains how Bcl-2 family members can regulate Mcl-1 ubiquitination (24), (T. Song, unpublished results). Therefore, TCTP and Mule might directly compete at the same Mcl-1 surface to dictate Mcl-1 fate. Yet, others have also suggested that Mcl-1 could act as a TCTP chaperone, thus maintaining cellular TCTP levels (21), highlighting the versatile nature of TCTP−Mcl-1 interaction.

TCTP owns a Bcl-2 Homology 3 (BH3) BH3-like motif (19) and is therefore among the few related proteins with anti-apoptotic properties (25). In the crystal structure of the TCTP BH3-like peptide in complex with Bcl-xL, the peptide binds to the BH3 binding groove of Bcl-xL (19). The TCTP BH3-like peptide adopts an α-helical conformation in the binding pocket of Bcl-xL, similar to other structures involving BH3 peptides and Bcl-2 proteins. However, while BH3 motifs are usually disordered or α-helical in the context of the free protein (26), the TCTP BH3-like motif is part of a β-sheet in full-length TCTP. Therefore, this suggests important changes in the structure and dynamics of full-length TCTP upon complex formation with Bcl-xL (19). Indeed, the BH3-like motif transition from β-sheet to α-helix is expected yet not characterized up to date. On the other hand, while the direct interaction between TCTP and Mcl-1 is documented on a functional level (8, 20, 21, 22), it remains unexplored from a structural perspective, which could give indications about how TCTP and Mcl-1 promote their relative cellular levels and functions.

Over the last 20 years, TCTP has become an attractive therapeutical target in cancers (2, 3, 4, 5, 6, 14, 27, 28). The antidepressant sertraline drug went into phase I/II clinical studies against Acute Myeloid Leukemia (AML), alone or in combination with Ara-C (29). Sertraline treatment allows for reducing TCTP levels and restoring p53 levels in tumor cells, and since sertraline was proposed to directly bind to TCTP, TCTP was suggested to be the cellular target of sertraline (10). Yet, we recently questioned the direct TCTP−sertraline interaction and proposed alternative modes of action of sertraline in tumor cells (30). Interestingly, AML often depends on high Mcl-1 levels (7, 31, 32). Moreover, in classical AML therapy, selective inhibitors of Mcl-1 are necessary to prevent the loss of Bcl-xL and subsequent apoptosis in healthy myeloid cells (33). Therefore, it is important to describe the TCTP−Bcl-xL and TCTP−Mcl-1 complexes from a structural perspective, including the suggested structural reorganization of TCTP, to draw rationales for the design of selective inhibitors.

In this study, we characterized the changes in TCTP structure and dynamics upon complex formation with the Bcl-2 family member Mcl-1 by using a combination of biophysical and biochemical methods (Nuclear magnetic resonance (NMR), SAXS, CD, SEC). In addition to revealing details about the major modification in the structure of full-length TCTP upon complex formation, we used peptidic approaches and molecular docking to highlight the important role of the non-canonical residue D16 in the TCTP BH3-like motif for the binding of TCTP to Bcl-2 family partners.

## Results

### The TCTP_BH3_ peptide binds in the BH3-binding groove of Mcl-1

**Figure 1:**
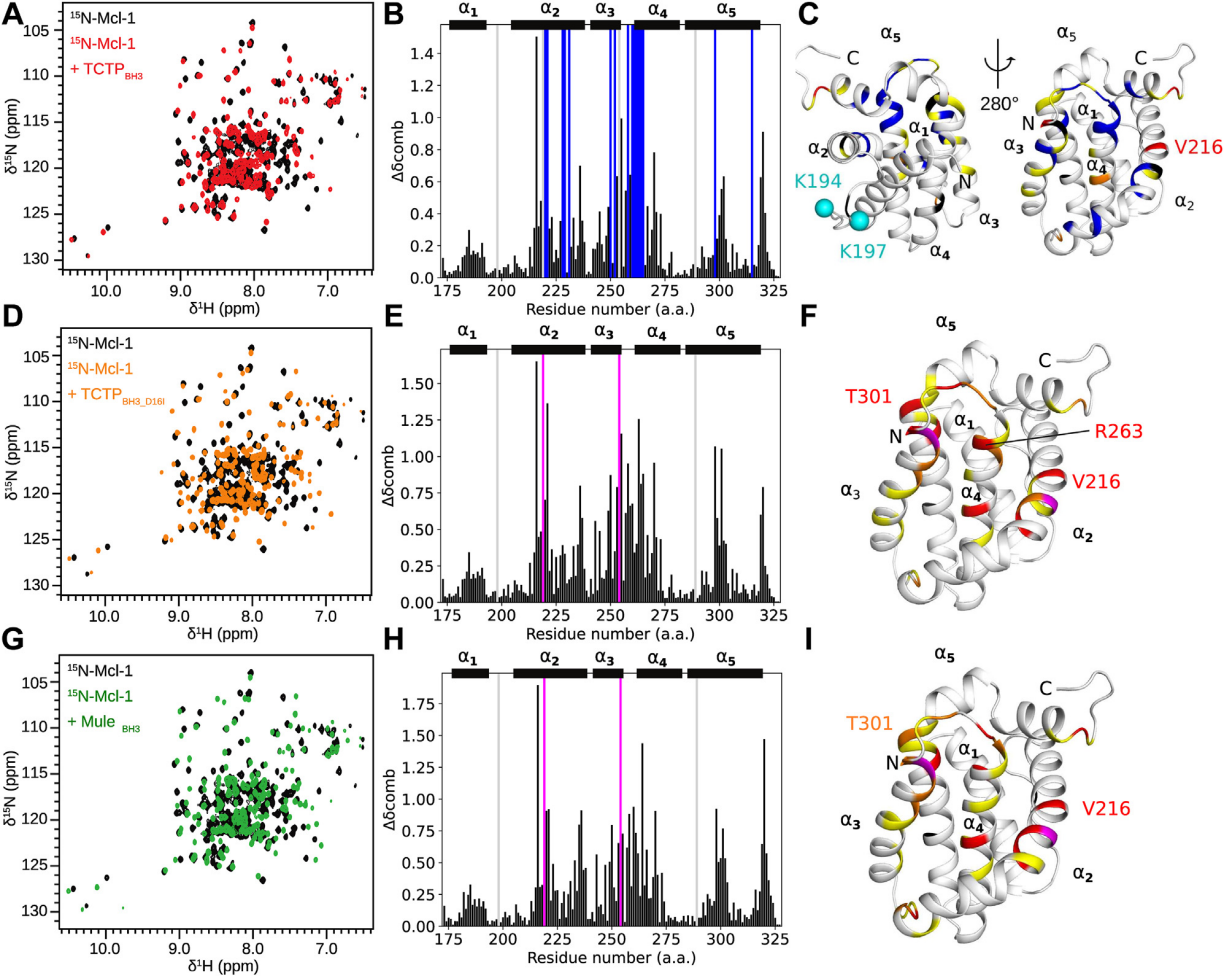
**NMR characterization of TCTP**_**BH3**_**and TCTP**_**BH3D16I**_**binding to Mcl-1.***A*, *D*, and *G*, overlay of ^15^N SOFAST-HMQC spectra from isolated ^15^N-Mcl-1 ΔPESTΔTM (Mcl-1) (100 μM, *black*) and in the presence of excess of unlabeled (*A*) TCTP BH3-like peptide (TCTP_BH3_) or (*D*) TCTP BH3-like peptide D16I mutant (TCTP_BH3D16I_) or (*G*) Mule BH3 peptide (MuleBH3). *B*, *E*, and *H*, combined ^1^H-^15^N chemical shift perturbations in the presence of (*B*) TCTP_BH3_ or (*E*) TCTP_BH3D16I_ or (*H*) MuleBH3. Exchange-broadened residues (*blue*), appearing residues (*magenta*), and unassigned residues in both free and bound states (*gray*) are highlighted. *C*, *F*, and *I*, Mapping of combined ^1^H-^15^N chemical shift perturbations in the presence of (*C*) TCTP_BH3_ or (*E*) TCTP_BH3D16I_ or (*H*) MuleBH3 (Δδcomb > 0.9 ppm, *red*; 0.9 ppm > Δδcomb > 0.65 ppm, *orange*; 0.65 ppm > Δδcomb > 0.4 ppm, *yellow*; Δδcomb < 0.4 ppm, *white*); exchange broadened residues (*blue*), appearing residues (*magenta*), and unassigned residues in both free and bound states (*black*) are highlighted on the NMR structure of Mcl-1 (79). Reported ubiquitination sites K194 and K197, located away from the BH3-binding groove, are highlighted (*cyan*) (37). Experiments were recorded at 950 MHz and 308 K in the following buffer: 50 mM MES pH 6.5, 50 mM EPPS, 50 mM NaCl, and 2 mM TCEP in 95% H_2_O/5% D_2_O. NMR, Nuclear magnetic resonance; TCTP, Translationally Controlled Tumor Protein.

There is currently no structural description available for the molecular interaction between TCTP and Mcl-1. Here, we first investigated by NMR spectroscopy if and where the TCTP BH3-like peptide TCTP_BH3_ could bind Mcl-1. Both NMR and circular dichroism analysis revealed that TCTP_BH3_ was essentially unfolded in the absence of Mcl-1 (Fig. S2, *B* and *C*). We then recorded ^15^N SOFAST-HMQC spectra of ^15^N-labeled Mcl-1 upon the addition of unlabeled TCTP_BH3_ up to a peptide: Mcl-1 molar ratio of 5:1 (Fig. 1*A*). Under these conditions, the formation of a Mcl-1−TCTP_BH3_ complex was very fast (<min) and clearly visible through the perturbation of many Mcl-1 NMR cross-peaks. Many of these cross-peaks were predominantly in the intermediate exchange regime, with signal vanishing near mid-titration and recovering only at high peptide concentration (Fig. S4*A*). Using 3D triple resonance experiments, we could assign 96.2% (152/158 residues) and 86.1% (136/158 residues) of free and bound Mcl-1 backbone resonances, respectively (File S1). The Gly-Pro sequence at the N-terminus, resulting from the Prescission cleavage site, and proline residues P198 and P289 were not assigned due to the lack of H-N signals. Additional unassigned residues for the apoprotein were G219 and F254 in helix α_2_ and in the loop connecting α_3_–α_4_, respectively. For the complex, unassigned residues were G219, V220, Q221, F228, Q229, and M231 in helix α_2_; M250, H252, and F254 in helix α_3_; V258 in the loop connecting α_3_–α_4_; T260, W261, G262, R263, I264, and V265 in helix α_4_; and L298 and F315 in helix α_5_. The higher number of unassigned residues in the complex is due to missing signals in the ^15^N SOFAST-HMQC spectrum, revealing changes in local dynamics. To further map the binding site of TCTP_BH3_, we computed the ^1^H-^15^N combined chemical shift perturbations between free and bound Mcl-1 (Fig. 1*B*). We colored the residues on the structure of Mcl-1 accordingly, and we highlighted those with dramatic signal extinction in blue (Fig. 1*C*). The largest perturbations, including chemical shifts and signal extinction, were consistently clustered within α-helical regions defining the BH3-binding groove of Mcl-1: helices α_2_, α_3_, α_4_ and in the α_3_–α_4_ loop (Fig. 1*C*). Of note, we found perturbations in helix α_5_ for residues L298 and T301, not located within the BH3-binding groove of Mcl-1. This is consistent with other reports about Mcl-1 and BH3 motif interactions (34, 35, 36), and it might indicate a conformational change in Mcl-1 upon complex formation with peptides. We also show the location of K194/K197 that are reported as Mule ubiquitination sites (Fig. 1*C*) (37). These lysines are not part of the BH3 binding region, suggesting that the TCTP_BH3_ binding interface and Mule ubiquitination sites do not overlap. Finally, we used the 2D line shape analysis software TITAN (38) to estimate a dissociation constant K_D_ of 7.9 ± 0.5 μM (with a dissociation rate k_off_ = 56 ± 2 s^−1^) for the TCTP_BH3_−Mcl-1 complex (see Experimental procedures). This value is consistent with the K_D_ of 12 μM reported by others using Isothermal Titration Calorimetry on the TCTP_BH3_ complex with Mcl-1 homolog Bcl-xL (19). Overall, we demonstrated that TCTP_BH3_ peptide binds to the BH3-binding groove of Mcl-1.

The presence of chemical exchange at the intermediate regime at the chemical shift time scale (μs-ms) is evidenced by the total extinction of NMR signals from Mcl-1 residues located at the interface in the complex in excess of peptide. In principle, such line broadening might originate either from the exchange between the free and the bound Mcl-1 (the estimated free Mcl-1 at 5 TCTP_BH3_ equivalents is ∼2% from TITAN analysis) or from the exchange between different binding modes within the Mcl-1−TCTP_BH3_ complex. Here we could not increase peptide concentration to further reduce the population of free Mcl-1. We also failed to produce recombinant ^15^N-labeled TCTP_BH3_, which could have revealed the dynamic properties of the peptide. However in the complex between full-length TCTP and Mcl-1 (*vide infra*), despite the difference in complex formation kinetics, we observed that the residues at the interface on Mcl-1 as well as the BH3 region of TCTP also suffered from extensive line broadening in excess of the other partner. Therefore, it seems plausible that the line-broadening observed for Mcl-1 with TCTP_BH3_ results from μs-ms exchange within the complex, likely revealing multiple binding modes interconverting at the μs-ms timescale. However additional contribution from free/bound exchange cannot be totally ruled out.

### Conserved TCTP D16 residue contributes to control TCTP BH3 binding to Mcl-1

**Figure 2:**
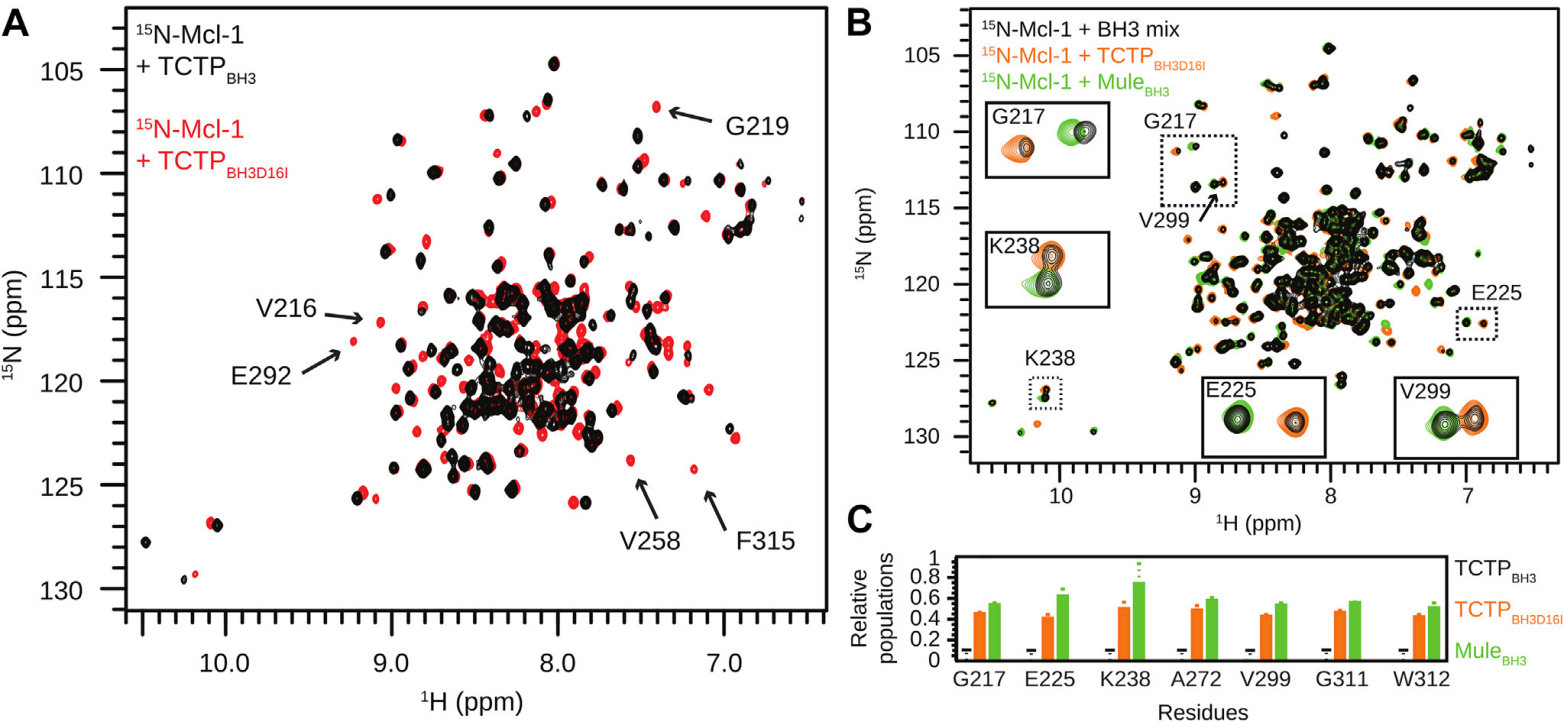
**Contribution of TCTP D16 residue in the binding mode to Mcl-1.***A*, overlay of ^15^N SOFAST-HMQC spectra from ^15^N-Mcl-1 ΔPESTΔTM (Mcl-1) (100 μM) in complex with TCTP_BH3_ (5 eq., *black*) or TCTP_BH3D16I_ (3 eq., *red*). *B*, overlay of ^15^N SOFAST-HMQC spectra from ^15^N-Mcl-1 in the presence of an equimolar mix of BH3 peptides (TCTP_BH3_, TCTP_BH3D16I,_ and MuleBH3; 5 eq. total, *black*) or with TCTP_BH3D16I_ (3 eq., *orange*) or MuleBH3 (3 eq., *green*) alone. *C*, relative population of ^15^N-Mcl-1 bound to TCTP_BH3_, TCTP_BH3D16I_, or MuleBH3. Experiments were recorded at 950 MHz and 298 K in the following buffer: 50 mM MES pH 6.5, 50 mM EPPS, 50 mM NaCl, 2 mM TCEP in 5% D_2_O/95% H_2_O. TCTP, Translationally Controlled Tumor Protein.

The BH3-like motif in TCTP has uncommon properties because it exerts anti-apoptotic functions (19). In contrast to canonical BH3 motifs, it contains a conserved aspartate residue (D16) (1) at the so-called position h1, while a hydrophobic residue is generally found at this position. Canonical BH3 peptides usually adopt a helical structure in complex with Bcl-2 proteins, and the residue at this position creates direct contact with the protein partner. In the case of the TCTP_BH3_−Bcl-xL structure (19), the side chain of D16 creates a contact with Q211. In order to assess the role of D16 in the binding mode of TCTP with Mcl-1, we designed the single-point mutant TCTP_BH3D16I_ peptide, where the canonical BH3 pattern was restored by changing aspartate to isoleucine (Fig. S2*A*). As for TCTP_BH3_, circular dichroism analysis showed that TCTP_BH3D16I_ was essentially unfolded in the absence of Mcl-1 (Fig. S2*B*). Then, we recorded ^15^N SOFAST-HMQC spectra of isolated ^13^C^15^N-Mcl-1 and upon addition of up to 3 equivalents of unlabeled TCTP_BH3D16I_ peptide (Fig. 1*D*). The complex between TCTP_BH3D16I_ and Mcl-1 was fully formed between 1 and 2 molar equivalents of the peptide. Under these conditions, all residues assigned in the apo Mcl-1 were clearly visible in the spectrum of bound Mcl-1, in sharp contrast with the complex with TCTP_BH3_. During the titration of Mcl-1 with TCTP_BH3D16I_, a significant portion of the NMR cross-peaks showed an intermediate-to-slow exchange regime between free and bound Mcl-1 states, characterized by visible and well-separated cross-peaks for the free and bound states near mid-titration, but small peptide-concentration-dependent chemical shift variations at the beginning/end of the titration (Fig. S4*B*). This indicates that the free/bound-state exchanging process is significantly slower than for TCTP_BH3_ (Fig. S4*A*). Using triple resonance experiments, all non-proline backbone residues of Mcl-1 in complex with TCTP_BH3D16I_ were assigned, including the residues in the BH3 binding groove (File S1). The sharp signals indicated a dramatic change in dynamics in the binding interface upon D16I mutation, enabling the recovery of all Mcl-1 NMR signals (Fig. 2*A*). We then computed the chemical shift perturbations (Fig. 1*E*) that we reported on the Mcl-1 structure (Fig. 1*F*). Peaks of residues within helices α_2_, α_3_, and α_4_ were largely shifted, confirming that the D16I mutant binds to the BH3-binding groove of Mcl-1. Taken together, the TCTP_BH3_ and TCTP_BH3D16I_ peptides have very similar binding interfaces on Mcl-1. Yet, the presence of aspartate at position h1 triggers extensive μs-ms motions at the interface, which are essentially quenched when isoleucine is present at position h1.

To compare the binding mode of the TCTP BH3 motif with a canonical BH3 peptide and to gain further insight into the relative roles of TCTP and Mule E3 ligase on Mcl-1, we designed the Mule_BH3_ peptide (Fig. S2*A*). As for peptides derived from TCTP BH3-like motif, circular dichroism analysis showed that Mule_BH3_ was essentially unfolded in the absence of Mcl-1 (Fig. S2*B*). We then carried out an NMR interaction experiment between ^15^N labeled Mcl-1 and up to 3 molar equivalents of Mule_BH3_ (Fig. 1*G*). As for TCTP_BH3D16I_, all expected signals, including those of the BH3 binding groove, were detectable for Mcl-1 and could be assigned (File S1). We monitored the shift of many Mcl-1 NMR cross-peaks, which were in intermediate-to-slow exchange regime during the titration (Fig. S4*C*), similar to TCTP_BH3D16I_. The complex between Mule_BH3_ and Mcl-1 was fully formed with 1 molar equivalent of peptide, in agreement with the reported dissociation constant K_D_ of 29 nM for the complex (34). Then, we confirmed from chemical shift perturbation analysis (Fig. 1*H*) that Mule_BH3_ binds to the BH3-binding groove of Mcl-1 (Fig. 1*I*). This result is in full agreement with the crystal structure of Mcl-1−Mule_BH3_ complex (PDB code: 5C6H). The strong signals for the BH3 binding groove indicate the absence of significant μs-ms dynamics at the Mule_BH3_−Mcl-1 interface, similar to TCTP_BH3D16I_, but in contrast to TCTP_BH3_.

NMR titration experiments revealed a slower free/bound state exchange regime for Mule_BH3_ and TCTP_BH3D16I_ peptides compared to TCTP_BH3_. To further explore the thermodynamics of the binding, we performed a competition binding experiment with the three peptides. We prepared an equimolar mix of each of TCTP_BH3_, TCTP_BH3D16I,_ and Mule_BH3_ (BH3 mix), and we added an excess of BH3 mix (5 eq.) with ^15^N labeled Mcl-1. We measured ^15^N SOFAST-HMQC spectra after 2 h incubation at room temperature (Fig. 2*B*). Based on the known spectra of Mcl-1 bound individually to each peptide (Fig 1, *A*, *D*, and *G*; File S1), we could identify seven residues as reporter signals that show distinct chemical shifts in the three complexes (G217, E225, K238, A272, V299, G311, W312). All these residues are away from the interface and did not experience significant signal intensity reduction upon peptide binding. For all reporter residues, when the three peptides were mixed, we could detect the spectral signatures of Mcl-1 in complex with TCTP_BH3D16I_ and Mule_BH3_ but not for the complex with TCTP_BH3_ (Fig. 2, *B* and *C*). This confirmed that the TCTP_BH3_ peptide has a significantly weaker affinity (at least 10 times weaker) for Mcl-1 than TCTP_BH3D16I_ and Mule_BH3_. Using the relative signal intensities of the reporter residues as a proxy of the relative populations of the complexes, we demonstrated that the canonical BH3 motifs Mule_BH3_ and TCTP_BH3D16I_ have similar affinity for Mcl-1 with relative populations of 55% and 45% (Fig. 2*C*). These results are fully in agreement with the nanomolar affinity of the Mule_BH3_−Mcl-1 complex (34) and the micromolar affinity of the TCTP_BH3_−Mcl-1 complex estimated in the previous section. Overall, the presence of an isoleucine residue at position h1 in TCTP increases the affinity of TCTP for Mcl-1 to a level similar to that of Mule_BH3_. This further indicates that Mcl-1 has a stronger affinity for the BH3 motif of Mule than that of TCTP. In conclusion, residue D16 of TCTP plays an important role in the control of affinity and interface dynamics of the TCTP−Mcl-1 complex.

### Structural models of Mcl-1 in complex with TCTP_BH3_ or TCTP_BH3D16I_

We next attempted to gain atomic details about the Mcl-1 complex with TCTP BH3-like peptide (TCTP_BH3_). We failed to obtain crystals of the complex. This may be due to its low affinity and/or internal intrinsic dynamics. As an alternative, we generated structural models through docking experiments, by using the HADDOCK web interface (39) and our NMR chemical shift perturbation data (see Experimental procedures).

**Figure 3:**
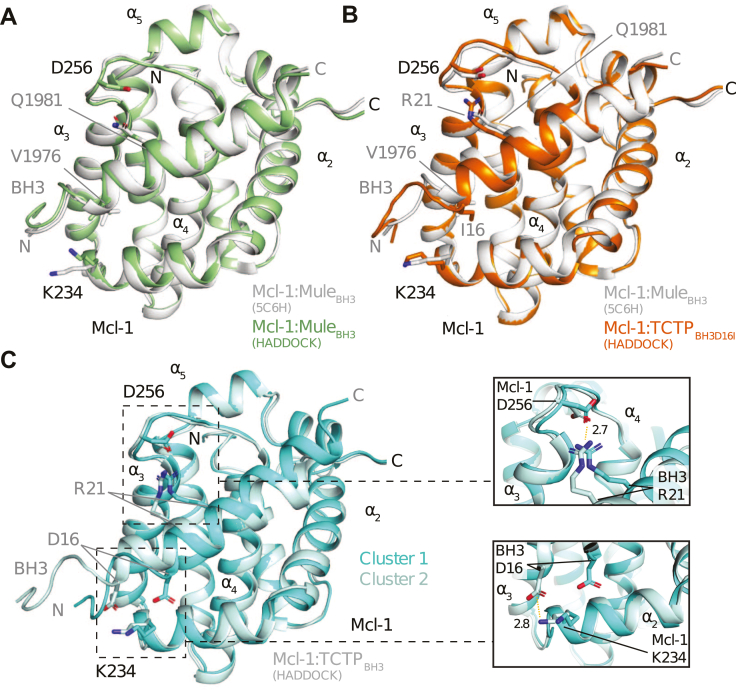
**Docking studies of Mcl-1 in complex with BH3 peptides using HADDOCK.***A*, alignment between the representative structure of the top-ranked cluster for Mcl-1−MuleBH3 and the experimental X-ray structure of Mcl-1−MuleBH3 complex (pdb code: 5C6H). *B*, alignment between the representative structure of the top-ranked cluster for Mcl-1−TCTPBH3D16I and the experimental X-ray structure of Mcl-1−MuleBH3 complex (PDB code: 5C6H). *C*, (*left*) alignment between the representative structure of clusters 1 and 2 for Mcl-1−TCTPBH3. (*right*) Close-up view of the electrostatic interactions between Mcl-1 D256 with TCTPBH3 R21, and Mcl-1 K234 with TCTPBH3 D16. Electrostatic interactions are highlighted (*dashed orange lines*). TCTP, Translationally Controlled Tumor Protein.

HADDOCK successfully generated a set of ranked clusters and representative structures for each target-ligand complex, with the HADDOCK score indicating the relative likelihood of the prediction. To validate the procedure, we compared the first-ranked cluster for the Mcl-1−Mule_BH3_ complex to its experimental crystal structure (PDB code: 5C6H) (Figs. 3*A* and S5*A*). The HADDOCK model showed a good agreement with the experimental structure of Mcl-1−Mule_BH3_ upon alignment of the entire complex (RMSD Cα = 0.488 Å). Upon alignment of bound Mcl-1 only, the RMSD computed between Mule_BH3_ in the HADDOCK model and the experimental structure was also reasonable (RMSD Cα = 1.186 Å). We observed a polar interaction between residues D256 (Mcl-1) and Q1981 (Mule) in both HADDOCK model and experimental structure. The NMR cross peak for residue Mcl-1-D256 was broad in the apoprotein, and the interaction resulted in a sharpening of its NMR signal, suggesting a reduction in internal dynamics upon BH3 binding (File S1).

The TCTP_BH3D16I_ peptide also adopts the canonical binding mode in the BH3 binding groove of Mcl-1, like Mule_BH3_. In the top-ranked cluster, TCTP_BH3D16I_ showed little difference from the Mcl-1−Mule_BH3_ complex and occupied the same hydrophobic cleft as Mule_BH3_ (Figs. 3*B* and S5*B*). Notably, the side chain of BH3-I16 occupies the same hydrophobic cleft in Mcl-1 as Mule-V1976. Mcl-1−TCTP_BH3D16I_ complex formation also resulted in a sharper Mcl-1-D256 NMR signal. Our HADDOCK model shows a salt bridge between Mcl-1-D256 and TCTP_BH3D16I_-R21. These results suggest that TCTP-R21 and Mule-Q1981 play similar roles in stabilizing Mcl-1-D256.

In the previous section, we highlighted that D16 impacts both the affinity and the interfacial dynamics of the TCTP−Mcl-1 complex. In order to understand the role of D16 in the BH3-like motif of TCTP, we docked a helical peptide containing the TCTP_BH3_ sequence onto Mcl-1, and we report the alignment of the two best-ranked clusters for the complex (Figs. 3*C* and S5*C*). In the top-ranked cluster, the BH3-like peptide adopts a canonical arrangement in the BH3-binding groove of Mcl-1 with D16 occupying a similar position as I16 in canonical BH3 peptides. In the second-ranked cluster, the α-helix formed by the BH3-like motif was shifted by 5.6 Å toward the N-terminus of the peptide. The h2, h3, and h4 residues occupy the same spatial positions as h1, h2, and h3 in the first-ranked cluster. Despite this shift, the salt bridge between TCTP_BH3_- R21 and Mcl-1-D256 remained intact in both clusters. This is consistent with previous mutagenesis studies, which showed that the R21A mutation in the TCTP BH3-like motif disrupts binding to Mcl-1 (21). Notably, the second cluster revealed that the side chain of Mcl-1-K234 forms a salt bridge with the side chain of TCTP_BH3_-D16. This suggests that when TCTP_BH3_ is in complex with Mcl-1, K234 could interact with D16 to create a unique non-canonical binding mode that is specific to wild-type TCTP and associated with the global 5.6 Å shift of the BH3 helix within the groove. This transition between canonical and non-canonical binding modes may occur at the microseconds to milliseconds timescale, as suggested by the line-broadening observed in NMR spectra. The NMR line-broadening observed for Mcl-1 residues F228 and M231, located around K234, with TCTP_BH3_, while they are not perturbed upon TCTP_BH3D16I_ binding, also supports the hypothesis of an extended Mcl-1−TCTP interface with TCTP_BH3_ compared to TCTP_BH3D16I_ (Fig. 1). Conversely, we propose that with the canonical TCTP_BH3D16I_ and Mule_BH3_ sequences, this conformational sampling is quenched because the hydrophobic residue at position h1 does not allow the formation of the salt bridge with K234.

### Comparison of full-length TCTP and TCTP_BH3_ peptide binding to Mcl-1

**Figure 4:**
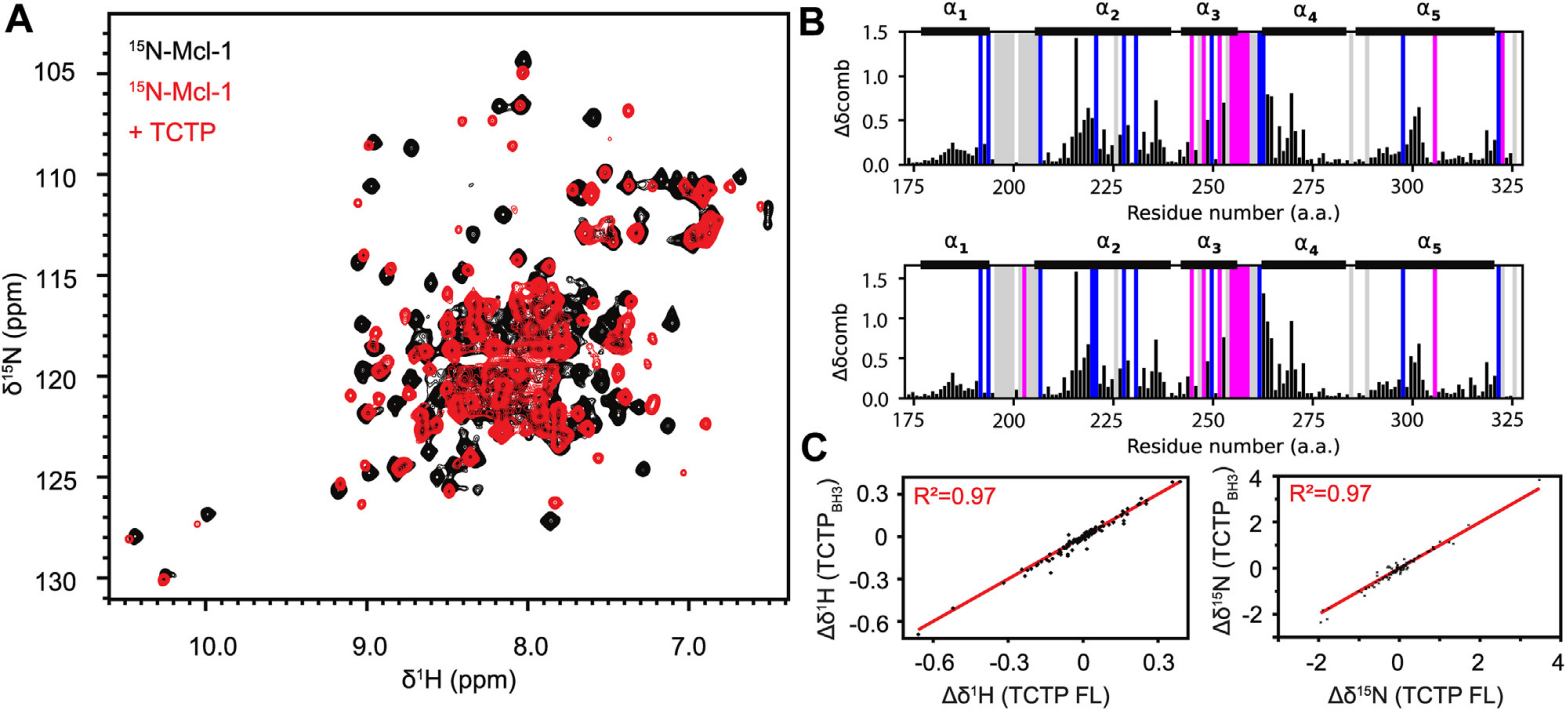
**NMR characterization of full-length TCTP binding to Mcl-1.***A*, overlay of ^15^N SOFAST-HMQC spectra from isolated ^15^N-Mcl-1 ΔPESTΔTM (Mcl-1) (100 μM, *black*) and in the presence of unlabeled FL-TCTP (2 eq., *red*) after 2 h incubation. *B*, alignment of combined ^1^H^−15^N chemical shift perturbations calculated between isolated ^15^N-Mcl-1 and in complex with TCTP BH3-like peptide (*top*) or FL-TCTP (*bottom*). Largest perturbations were found for residues V216, D218, G219, D236, R263, I264, V265, F270, F273, R300, T301, and K302. Disappearing (K194, R207, V220, Q221, F228, M231, M250, G262 and L298; *blue*) and appearing (S245, R248, H252, V253, S255, D256, G257, V258 and T259; *magenta*) residues are highlighted. *C*, correlation of ^1^H (*left*) and ^15^N (*right*) chemical shifts perturbations from Mcl-1 in complex with FL-TCTP or TCTP BH3-like peptide. Experiments were recorded at 950 MHz and 308 K in the following buffer: 50 mM EPPS pH 8, 50 mM MES, 50 mM NaCl, 2 mM TCEP in 5% D_2_O/95% H_2_O. NMR, Nuclear magnetic resonance; TCTP, Translationally Controlled Tumor Protein.

In order to compare the binding profiles of full-length TCTP (FL-TCTP) and the truncated TCTP_BH3_ peptide, we next characterized the interaction between FL-TCTP and Mcl-1 by NMR. We recorded ^15^N SOFAST-HMQC spectra of isolated ^15^N-labeled Mcl-1 and upon addition of 2-fold excess of unlabeled FL-TCTP (Fig. 4*A*). In sharp contrast with Mcl-1−TCTP_BH3_, the formation of the Mcl-1−FL-TCTP complex was slow (∼hrs) as judged from the slow appearance of cross-peaks corresponding to the complex and disappearance peaks of apo Mcl-1. In our hands, the stability of the Mcl-1−FL-TCTP complex in solution required higher temperatures and alkaline pH, which is reminiscent of previous observations reported for the TCTP−Bcl-xL complex (19). In the ^15^N SOFAST-HMQC spectrum of Mcl-1 in complex with FL-TCTP, the visible NMR cross-peaks were generally broader than those of TCTP_BH3_, in agreement with a complex of larger molecular size (Fig. S6*A*). As for TCTP_BH3_, several cross-peaks disappeared in the ^15^N SOFAST-HMQC spectrum of Mcl-1 bound to full-length TCTP for residues in the BH3 binding groove (Fig. 4*B*). This strongly suggests that the conformational exchange at intermediate timescales (μs-ms) observed with TCTP_BH3_ is also present in the complex with FL-TCTP. With FL-TCTP, the exchange timescale between the free and the bound state is (very) slow, suggesting that the line broadening observed for Mcl-1 is likely not due to free/bound exchange but rather to the enhanced interface dynamics hypothesized in the previous section. We attempted to assign *de novo* the ^15^N SOFAST-HMQC spectrum of Mcl-1 in complex with FL-TCTP, but we could not obtain exploitable 3D triple resonance spectra.

To circumvent these difficulties, we used the same conditions of temperature and alkaline pH required to assemble Mcl-1−FL-TCTP complex to record the ^15^N SOFAST-HMQC spectrum of Mcl-1 in complex with TCTP_BH3_ peptide. The ^15^N SOFAST-HMQC spectra of Mcl-1 bound to TCTP_BH3_ or FL-TCTP were highly resembling in terms of chemical shift profile (Fig. S6*B*). Therefore, we could easily transfer the chemical shift assignment of Mcl-1 from the complex with TCTP_BH3_ peptide to the complex with FL-TCTP. Residue-specific information was thus obtained on ^1^H, ^15^N chemical shift perturbation, signal appearance (magenta) and disappearance (blue) for Mcl-1 in complex with TCTP_BH3_ peptide or TCTP protein (Fig. 4*B*) and the ^1^H and ^15^N chemical shifts of Mcl-1 in complex with TCTP_BH3_ plotted *versus* the complex with FL-TCTP (Fig. 4*C*). Taken together, ^1^H and ^15^N chemical shift analysis revealed that FL-TCTP binds the BH3-binding groove of Mcl-1 very similar to the TCTP_BH3_ peptide, with a similar binding interface and conformational exchange at the molecular interface. Moreover, these results suggest that the interacting region in TCTP is limited to the BH3-like motif and does not involve other regions of TCTP. While sharing a similar binding mode to Mcl-1, TCTP_BH3_ peptide and FL-TCTP protein display a striking difference in their association kinetics with Mcl-1. The slow (∼ hrs) formation of the complex with FL-TCTP strongly suggests a rate-limiting structural reorganization of TCTP prior to complex formation.

To compare the relative affinity of FL-TCTP and TCTP_BH3_ for Mcl-1, we carried out a competition experiment by NMR (Fig. S7). First, the ^15^N labeled FL-TCTP−Mcl-1 complex was prepared, which led to the disappearance of most signals in ^1^H-^15^N correlation spectra for TCTP. Then, TCTP_BH3_ peptide was added, and most free FL-TCTP signals were recovered (Fig. S7*A*). In the reciprocal experiment, the TCTP_BH3_−Mcl-1 complex was prepared, leading to chemical shift perturbation and line broadening of 1D methyl signals for Mcl-1. Then, FL-TCTP was added and no change in spectral signatures from Mcl-1 bound to TCTP_BH3_ could be observed after equilibration time, neither for methyl signatures of free FL-TCTP (Fig. S7*B*). This suggests that the TCTP BH3 peptide effectively competes with full-length TCTP for the same binding site on Mcl-1, exhibiting a higher apparent affinity. Additionally, the conformational alteration in TCTP upon binding to Mcl-1 is reversible. Due to the very slow kinetics of complex formation with FL-TCTP, we did not attempt to precisely measure the affinity of the complex.

### Conformational change in TCTP upon binding Mcl-1

**Figure 5:**
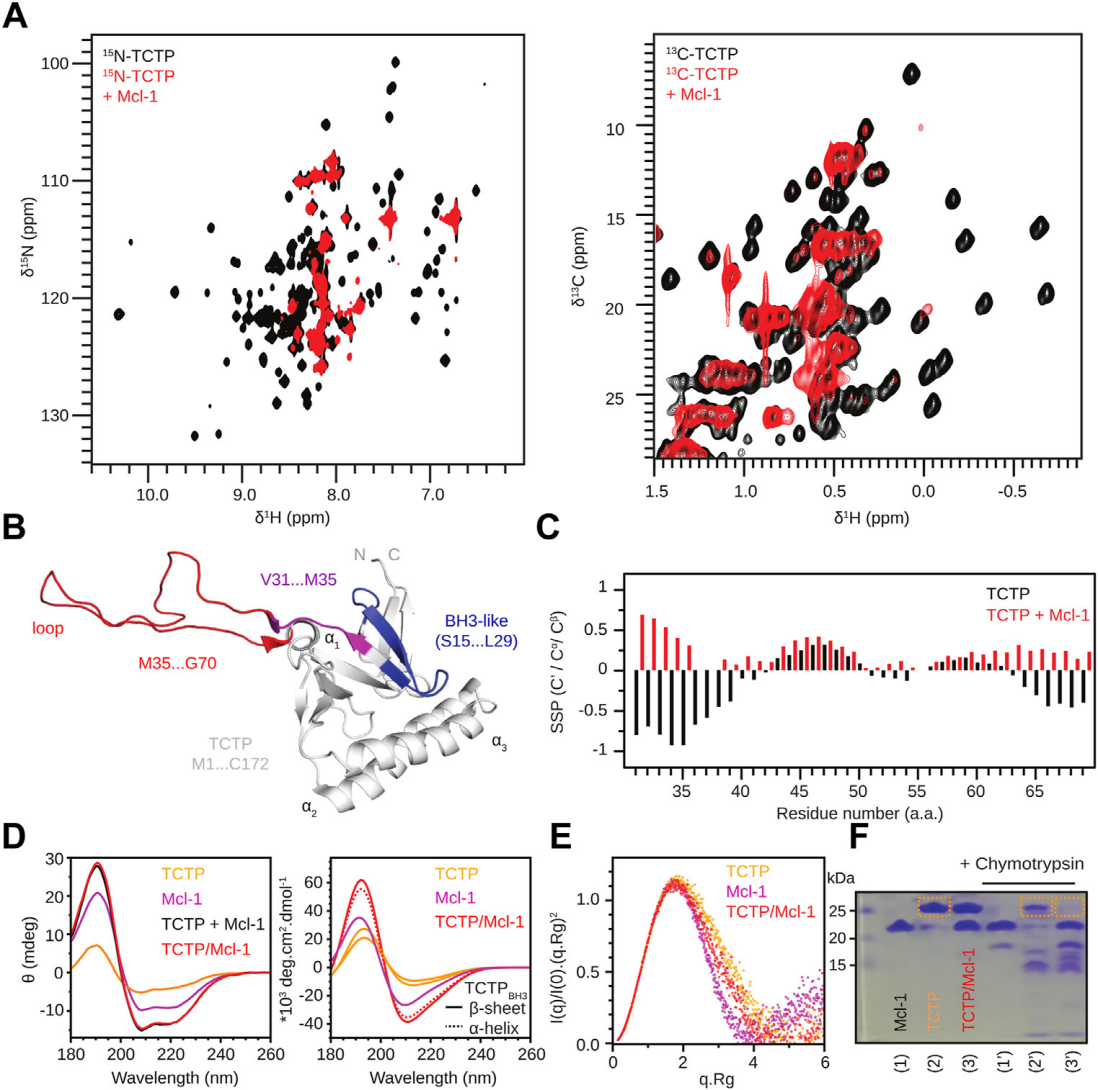
**Characterization of molten-globule state of TCTP in complex with Mcl-1.***A*, overlay of (*left*) ^15^N and (*right*) ^13^C SOFAST-HMQC spectra from isolated ^15^N-TCTP (100 μM, *black*) and in the presence of unlabeled ΔPESTΔTM (Mcl-1) (2 eq., *red*) after 2 h incubation. Experiments were recorded at 950 MHz and 278 K in the following buffer: 50 mM EPPS pH 8, 50 mM MES, 50 mM NaCl, 2 mM TCEP in 5% D_2_O/95% H_2_O. *B*, the region of TCTP (V31 to G70), for which NMR backbone signals could be assigned, is represented on the NMR structure of the protein (42). The long flexible loop (*red*), the BH3-like motif (*blue*), and its C-terminal extension visible by NMR (*magenta*) are highlighted. *C*, Secondary Structure Propensity (SSP) (40) computed for isolated TCTP (*black*) and in complex with Mcl-1 (*red*). Positive values indicate α-helix propensity whereas negative values indicate β-strand propensity. *D*, (*left*) Far-UV CD experiments on isolated TCTP (50 μM, *orange*), isolated Mcl-1 (50 μM, *magenta*), sum of the two CD curves (*black*) and experimental CD curve of the equimolar mix of the two proteins (50 μM each, *red*) after 2 h incubation. Experiments were performed at 308 K in 5 mM phosphate buffer pH 8 and 100 μM TCEP to ensure data reliability at low wavelengths. (*right*) Theoretical prediction of CD curves using BeStSel (74) for native TCTP (*orange*), TCTP with BH3-like motif as α-helix (*dashed*, *orange*), Mcl-1 (*magenta*). The sum of Mcl-1 and native TCTP curves (*red*) or with BH3-like motif as α-helix (*dashed*, *red*). *E*, normalized Kratky plots for TCTP (*orange*), Mcl-1 (*magenta*), and the dominant heterodimeric TCTP−Mcl-1 complex (*red*). *F*, limited proteolysis experiments using chymotrypsin protease. Isolated Mcl-1 (500 μM), TCTP (500 μM), and an equimolar mixture were incubated overnight at 308 K in 50 mM EPPS pH 8, 50 mM MES, 50 mM NaCl and 2 mM TCEP with or without chymotrypsin (5 μg ml^−1^). *Dashed line rectangles* highlight the band for native TCTP in relevant conditions. TCTP, Translationally Controlled Tumor Protein.

In order to characterize the TCTP−Mcl-1 complex from the TCTP perspective, we recorded ^15^N and ^13^C SOFAST-HMQC spectra of isolated ^15^N-^13^C-labeled TCTP and upon complex formation with unlabeled Mcl-1 (Fig. 5*A*). In both spectra of isolated TCTP, all cross-peaks were well dispersed in both ^1^H and ^15^N or ^13^C dimensions, indicating that the visible part of the protein is well folded in our conditions. In a previous study, we reported Secondary Structure Propensity (SSP) (40) calculated from ^13^Cα, ^13^Cβ, and ^1^Hα chemical shifts using the same TCTP construct and nearly identical experimental conditions (41), which was in good agreement with the solution structure of full-length TCTP (42). After Mcl-1 was added and equilibrium was reached (2 eq., 2 h), most cross-peaks from the globular domain of TCTP became invisible due to the complex formation, while strong cross-peaks remained in spectral regions characteristic of disordered residues. Using 3D triple resonances experiments, these visible cross-peaks were easily assigned to the continuous stretch of residues V31 to V70, which encompasses the long internal loop and β-strands β_4_ and β_5_ in the structure of the free protein (Fig. 5*B*). We further analyzed ^13^C backbone chemical shifts using the SSP approach to assess secondary structure changes in the bound TCTP segment visible by NMR (Fig. 5*C*). Positive and negative SSP values reveal propensities for α-helical and β-strand conformations, respectively. When comparing SSPs for each residue, we observed that values remained unchanged for the loop central region from residue E40 to T65 between free and bound states, suggesting that the loop remains fully disordered in the complex. By contrast, residues in the segments from residues V31 to R38 and residues V66 to V70 underwent an inversion in SSP, suggesting a structural transition from β-strand to α-helical conformational propensity. In the free protein, they encompass the β_4_ and β_5_ strands that form a β-sheet at the bottom of the loop. This β-sheet breaks upon complex formation with Mcl-1, and residues V31-R38 and V66-V70 become largely flexible, with some helical propensity. Notably, the BH3-like region in TCTP (S15-L29) remained invisible in these NMR experiments, and therefore the transition from β-sheet to α-helix could not be directly evaluated by NMR. The helical propensity observed for neighboring residues (after V31) may reflect the extension of the putative BH3 helix beyond the BH3 motif when bound in the Mcl-1 groove.

The most striking feature is the disappearance of most NMR signals for TCTP residues beyond the V31-V70 region. The absence of signals for the BH3 motif is likely associated with the extensive μs-ms dynamics at the interface, similar to what was observed on the Mcl-1 side, and involving an exchange between canonical and non-canonical binding modes as suggested from docking experiments. However, vanishing signals for residues 1 to 14 and after 71, that is, the core protein region, needs to be explained. Severe NMR line-broadening can be expected for large protein complexes or in the case of internal dynamics. We therefore complemented our NMR data with additional structural techniques.

We first relied on far-UV (180–260 nm) Circular Dichroism (CD) spectroscopy to evaluate overall secondary structure changes by formation of the TCTP−Mcl-1 complex with respect to isolated proteins (Fig. 5*D*). The far-UV CD spectrum of isolated TCTP contains contributions from α-helix, β-strand, and random coil elements as expected from the crystal and NMR structures of the protein (42, 43). The far-UV CD spectrum of Mcl-1 only contained the contributions of α-helices in full agreement with its 3D structure. The experimental CD curve for the complex was compared to the sum of the CD curves for the two isolated proteins TCTP and Mcl-1. The two curves were strikingly similar, indicating that the secondary structure content in TCTP−Mcl-1 complex is preserved compared to isolated proteins. Of note, the putative β-strand to a α-helix transition for the BH3-like motif in TCTP is likely not to be seen herein, since it contributes to less than 5% of total residues in TCTP−Mcl-1 complex. Overall, CD analysis indicates limited changes in TCTP and Mcl-1 secondary structure upon complex formation.

### Quaternary structure of the FL-TCTP−Mcl-1 complex

**Figure 6:**
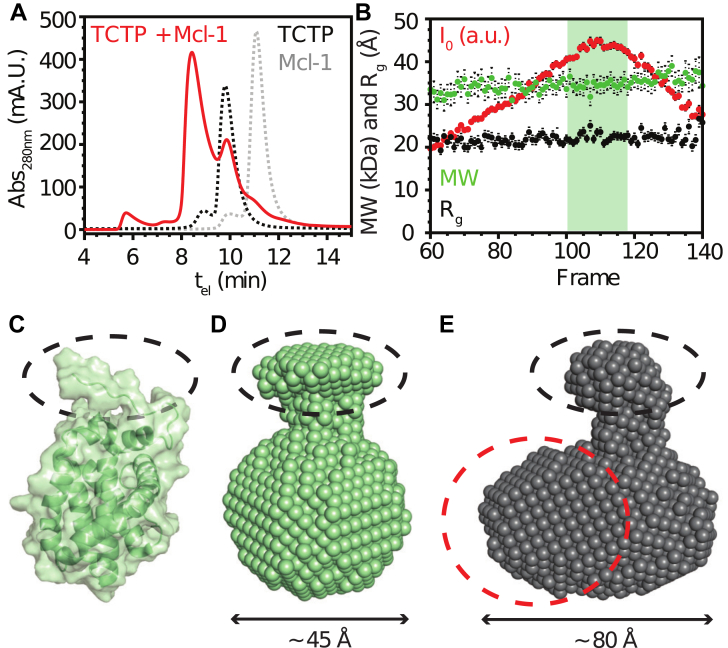
**Quaternary structure of TCTP−Mcl-1 complex from SEC-SAXS experiments.***A*, SEC profiles of isolated full-length TCTP (500 μM, *dashed black line*), isolated ^15^N-Mcl-1 ΔPESTΔTM (Mcl-1) (500 μM, *dashed gray line*), and an equimolar mix of TCTP and Mcl-1 after 2 h incubation (*red*). *B*, SAXS-derived parameters for TCTP−Mcl-1 complex. Scattering intensity I0, the molecular weight (MW), and the radius of gyration (Rg) are shown for the different frames along the SEC dimension. *C*, surface representation of Mcl-1 NMR structure (PDB code 2MHS, (79)). *D*, molecular envelope of free Mcl-1 derived from SAXS analysis. *E*, molecular envelope of free Mcl-1−TCTP complex derived from SAXS analysis. The plausible location of TCTP is highlighted as a *dashed red circle*. For (*C*), (*D*), and (*E*), the structural signature of Mcl-1 visible as a protuberance pointing out from the globular domain is highlighted as a *dashed black ellipse*. Experiments were performed at 308 K in the following buffer: 50 mM CHES pH 9, 50 mM NaCl, and 2 mM TCEP. TCTP, Translationally Controlled Tumor Protein.

In order to evaluate the stoichiometry of the TCTP−Mcl-1 complex, and hence its molecular size, we performed SEC-SAXS experiments. In SEC experiments, elution time was longer for isolated Mcl-1 (11.3 min) than for isolated TCTP (9.8 min), consistently with the molecular weights of 17.9 kDa for Mcl-1 and 19.6 kDa for TCTP. Upon complex formation, a major molecular species with higher apparent molecular weight eluted first (8.5 min), while residual free TCTP and Mcl-1 could still be observed (Fig. 6*A*), revealing incomplete complex formation. We next analyzed SAXS frames corresponding to the major species, presumably the TCTP−Mcl-1 complex (Fig. 6*B*). We found a molecular weight of 34.7 ± 1.9 kDa and a radius of gyration (R_g_) of 2.22 ± 0.09 nm. We expected a molecular weight of 37.4 kDa for the heterodimeric TCTP−Mcl-1 complex. Considering that the experimental value might be underestimated because residual quantities (<5%) of free TCTP co-elutes with the complex, our finding is consistent with a heterodimeric (1:1) TCTP−Mcl-1 complex. In addition, the experimental R_g_ of 2.10 ± 0.08 nm is compatible with a ∼35 kDa globular protein complex. We concluded that the TCTP−Mcl-1 complex is predominantly heterodimeric in solution.

We further exploited SEC-SAXS data by computing molecular envelopes using the Primus software DAMMIF. We identified a potential structural signature for isolated Mcl-1 that we highlighted on the structure of the protein (Fig. 6*C*) and the computed envelope (Fig. 6*D*). Interestingly, we could also detect this signature in the molecular envelope of the TCTP−Mcl-1 complex, suggesting a localization for Mcl-1 and thus TCTP in the heterodimeric complex (Fig. 6*E*). Overall, the TCTP−Mcl-1 complex had a prolate shape with a tightly packed organization, which confirmed the 1:1 binding stoichiometry. More details were not evaluated because residual quantities (<5%) of free TCTP co-eluted with the complex in SEC, making it difficult to obtain deconvoluted SAXS data with the optimal signal-to-noise ratio. Since density was missing for the long internal loop of TCTP in the molecular envelope of the complex, this region may retain its flexible character in the complex.

Molecular envelope computation poorly captures the presence of flexible regions, which are better detectable by the normalized Kratky analysis. We found that the Kratky plot for isolated Mcl-1 had a typical sine-bell shape that represents a globular, well-folded protein (Fig. 5*E*). The plot for isolated TCTP depicts a globular domain, but intensity values decreased slower with respect to the diffusion angle compared to the Mcl-1 plot. This is a signature of an extended segment, which can be correlated to the TCTP loop region that is absent in Mcl-1. Strikingly, the Kratky plot of the TCTP−Mcl-1 complex is highly similar to the mean of curves from isolated proteins, indicating that TCTP retains a packed tertiary structure in complex with Mcl-1, with the exception of the TCTP loop region that is flexible in free and bound states. This supports the earlier NMR analysis, leading us to conclude that TCTP and Mcl-1 form a closely associated heterodimeric complex. Within this complex, both proteins maintain characteristics related to their respective tertiary structures in the free state.

### Molten-globule properties of TCTP in complex with Mcl-1

NMR analysis revealed sharp signals for Mcl-1 in the complex but extensive line-broadening for TCTP. This indicates that TCTP and Mcl-1 have significantly different dynamic behaviors in the complex. Yet, SEC-SAXS analysis revealed a predominant 1:1 complex in the solution. This indicates that line-broadening in TCTP cannot be accounted for by the formation of large particles. To conciliate all data, we hypothesized that line-broadening in TCTP could originate from the destabilization of the core domain of TCTP into a so-called molten-globular state. Molten-globular states are compact conformational states with tertiary structures that fluctuate at the intermediate-to-slow timescales (μs-ms), while secondary structure elements remain essentially preserved. SEC-SAXS and CD analysis were indeed consistent with a compact nature of TCTP−Mcl-1 with well-formed secondary structures. As a consequence of extensive internal structural fluctuations in the molten-globule state, peptide bonds may be exposed to solvent while they would not be in the native state. Therefore, we performed limited proteolysis experiments with chymotrypsin to evaluate if the globular domain of TCTP could be more sensitive to proteolysis upon complex formation (Fig. 5*F*). We first assessed the stability of isolated proteins and complexes over time, without chymotrypsin addition, by SDS-PAGE. Then, we obtained full digestion profiles using high chymotrypsin concentration (100 μg ml^−1^). Finally, limited proteolysis with chymotrypsin was carried out (5 μg ml^−1^). For isolated Mcl-1, the limited digestion profile consisted of a major band corresponding to the undigested protein and a minor band corresponding to one digestion product. For isolated TCTP, a major band corresponding to the undigested protein was also seen along with multiple minor bands for digestion products. For the TCTP−Mcl-1 complex, a main band was observed for undigested Mcl-1, while the band corresponding to undigested TCTP disappeared, indicating that TCTP was fully digested. This suggests that peptide bonds are more exposed to solvent in bound TCTP. Overall, we demonstrated that the globular domain of TCTP in complex with Mcl-1 is characteristic of molten-globule states with preserved secondary structure content and a preserved tertiary structure that displays fluctuations at intermediate-to-slow timescales (μs-ms).

## Discussion

Previous studies have shown that the interaction between TCTP and Mcl-1 can impact their cellular stability (20, 21, 22). It was proposed that Mcl-1 acts as a stabilizing factor for TCTP (21), while TCTP reduces the degradation of Mcl-1 (20). During parasite infection, TCTP safeguards Mcl-1 from degradation by the E3 ubiquitin ligase Mule across multiple cell lines (22). The wide range of TCTP partners and biological functions raises questions about its structural adaptability (1). In this study, we aimed to elucidate the structural plasticity in TCTP and to highlight its role in the interaction with Mcl-1.

We have shown that TCTP binds to the BH3-binding groove of Mcl-1 through its BH3-like motif. The TCTP BH3-like motif promotes conformational exchange at the binding interface with Mcl-1 on a timescale of microseconds to milliseconds. A mutation to isoleucine at the non-canonical D16 residue at position h1 in the TCTP BH3-like motif restored the canonical BH3 pattern, abrogated the specific dynamics at the interface, and enhanced binding to Mcl-1. The crystal structure of Bcl-xL−TCTP_BH3_ showed a very similar molecular recognition, yet no interfacial dynamics were reported (19). The N-terminal extremity of the TCTP BH3-like motif is crucial for TCTP anti-apoptotic activity as demonstrated by the failure of a hybrid Bax/TCTP peptide to enhance the anti-apoptotic properties of Bcl-xL or to rescue the haploinsufficient phenotype of TCTP^+/^**^−^** thymocytes (19). This points to a potential role of D16 to promote the anti-apoptotic activity of TCTP, which remains to be established experimentally. Previous research also reported the importance of the R21 residue in TCTP interaction with Mcl-1 and Bcl-xL, with TCTP R21A mutant showing reduced binding to both partners (19, 21). Consistently, our structural model revealed electrostatic interactions between the R21 side chain and the Mcl-1 D256 residue in the α_3_α_4_ loop. In the Bcl-xL complex, R21 interacts with E129 and D133, similarly to its interaction with D256 in Mcl-1. This indicates that the loss of an intermolecular salt bridge in the R21A mutant may be sufficient to disrupt the binding of TCTP to Bcl-2 family partners. In the Mcl-1 protein, the mutation of K276 to valine disrupts the formation of the TCTP-Mcl-1 complex, while still preserving interactions with Bim and Bax (20). K276 forms an internal salt bridge with D218 and is located on the opposite side of helix α_2_ from the BH3 binding groove. Upon TCTP binding, the NMR shifts for the K276 backbone remain unchanged, suggesting that K276 does not play a direct role in the interface. However, the mutation that disrupts the K276-D218 salt bridge could potentially displace helix α_2_ at the BH3 binding groove, which may subsequently impact TCTP binding.

Mcl-1 degradation is controlled by E3 ligases such as Mule, SCFβ-TrCP, SCFFbw7, and Trim17, with Mule being the major one (24, 37, 44). The BH3 domain of Mule interacts with the BH3 binding groove of Mcl-1, leading to its degradation (37, 44, 45). This process is regulated by a group of proteins known as BH3-only proteins, which include Bak, Bim, Bid, Puma, and Noxa (45). A BH3 peptide from Bim can prevent Mule interaction with Mcl-1 in cells (44). We evaluated if TCTP could displace Mule at the Mcl-1 binding site, but competition experiments showed the TCTP BH3 peptide had weaker affinity than Mule BH3 peptide for Mcl-1, indicating that TCTP is unlikely to compete unless it is present at high concentration. Notably, TCTP is one of the most abundant proteins in eukaryotes (46) and is present at much higher levels in cancer cells than in Mule. This would preserve the possibility of competition in tumor cells.

The TCTP−Mcl-1 complex forms when the TCTP BH3 region docks onto Mcl-1. Within the complex, the TCTP globular domain is destabilized into a molten globule state. Given the similarity of binding mode with Bcl-xL, TCTP may also adopt this state in the TCTP−Bcl-xL complex. This hypothesis is supported by the findings of Susini *et al.* (43) who proposed that full-length TCTP may insert into the mitochondrial membrane by binding to Bcl-xL through the use of α-helices α_2_ and α_3_, which have structural homology with Bax α-helices α_5_ and α_6_. The molten globule state of TCTP may facilitate the insertion of TCTP−Bcl-xL complexes into membranes as molten globule states are known to enable protein insertion into membranes (47, 48, 49). We have also shown that the molten globule state of TCTP is more prone to proteolysis *in vitro*. In cells, chaperones often target these partially disordered or misfolded states for refolding or degradation. Interestingly, the small chaperone HSP27 has been shown to stabilize TCTP levels in prostate cancer cells by preventing its proteasomal degradation (13, 27). Future studies may investigate whether HSP27 can specifically target and stabilize TCTP molten globule state.

We found that TCTP BH3 peptide has a stronger binding to Mcl-1 compared to full-length TCTP. An *in vitro* GST pull-down assay showed that a TCTP construct missing the first ten residues strongly binds Mcl-1 (20). The first ten amino acids of TCTP contribute to the folding of TCTP into the β-tent (1) through multiple H-bond formation. The removal of these residues likely disturbed the overall TCTP structure, leading to the exposure of the BH3 region in the truncated form (20). This increased binding upon N-terminal truncation and the slow kinetics of TCTP−Mcl-1 complex formation observed by NMR suggests that TCTP may have conformational changes prior to binding with Bcl-2 family partners. This raises the question of a potential activation of TCTP in the cellular environment to regulate its anti-apoptotic function. TCTP can be easily phosphorylated by the Plk-1 kinase (50). We recently showed that TCTP does not show major conformational change upon phosphorylation at position S46 by Plk-1 (30), excluding S46 phosphorylation as a trigger of TCTP conformational activation. Bid is a well-known BH3-only protein that is activated by the caspase-8 protease (51, 52). In Bid, the BH3 region adopts an α-helical structure that becomes available for interaction with Bax and Bcl-2 in its truncated form t-Bid after caspase-8 mediated proteolytic cleavage (51, 52). TCTP being another BH3-only protein involved in apoptosis, its activation through caspase cleavage has already been considered (53, 54). In brief, a C-terminally cleaved TCTP is able to bind Apaf-1, but the TCTP sequence does not contain any known caspase enzymatic site, cannot be cleaved by recombinant caspase-3, and protease inhibitors could not help identify the involved protease activity for TCTP activation (53, 54). Further investigations are needed to determine if the TCTP BH3 region is activated through post-translational modifications such as phosphorylation, cleavage, or binding to other partners. We hypothesize that the TCTP BH3-like motif being buried in its globular domain may correspond to normal cell functioning, but that its activation may contribute to tumorigenesis by exposing its BH3 to partners.

TCTP has become an attractive drug target for cancer and other diseases as it interacts with many other proteins (1). Blocking these interactions may be a therapeutic strategy, as demonstrated by the drug sertraline, which prevents TCTP from binding to MDM2 to restore high levels of p53 (10). Small compounds targeting TCTP complexes with Mcl-1 or Bcl-xL may also be effective in reducing TCTP pro-survival properties. Although there are no obvious binding pockets in the TCTP globular structure, *in silico* drug screening has identified promising compounds (55, 56, 57). Our research has uncovered the biophysical properties of TCTP in complex with Mcl-1. We observed that full-length TCTP exhibits slow binding to Mcl-1, whereas the BH3 peptide binds rapidly. This observation suggests the existence of an uncommon, rate-limiting conformational switch that facilitates TCTP binding to Mcl-1. Small compounds that either prevent or facilitate this event could have the potential to control TCTP ability to regulate apoptosis through its interactions with Bcl-2 family partners. Additionally, compounds that affect TCTP stability in a molten globule state could impact the protein cellular stability and may offer an alternative to reducing TCTP levels in tumors, compared to the antisense oligonucleotide strategy (13).

In conclusion, our structural analysis has uncovered that TCTP adopts a molten-globule state in complex with Mcl-1. Our results establish the structural versatility of TCTP, which lays the foundation for understanding how it can interact with different partners and achieve its various biological functions (1). Further research is needed to understand the impact of the molten-globule state on protein-protein interactions. Our study broadens knowledge of TCTP versatility and offers new avenues for TCTP-targeted drug development.

## Experimental procedures

### Expression and purification of proteins

#### Production of full-length human TCTP

The details of TCTP production and purification were published (41) for ^15^N and/or ^13^C labeled TCTP protein. The protein sequence is given in the Supplementary material (Fig. S1).

#### Production of human Mcl-1

The plasmid pET15b (Amp^R^) encoding human Mcl-1 (UniProtKB: Q07820) with its PEST domain (ΔPEST) and its C-terminal transmembrane segment (ΔTM) deleted in fusion with N-terminal His_6_-MBP tag was used to express a soluble form of Mcl-1 (ΔPESTΔTM), as described in (58). A Prescission cleavage site was present between His_6_-MBP and Mcl-1. The protein sequence after cleavage is given in the Supplementary material (Fig. S1). Unlabeled Mcl-1 ΔPESTΔTM was expressed in *Escherichia coli* BL21 Rosetta (DE3) pLysS grown in rich medium 2xYT. Isotopic labeling was done using minimal medium M9 supplemented with ^15^N-labeled NH_4_Cl (1 g.L^−1^) and ^12^C-α-D-glucose (^15^N-labeling) or ^13^C-α-D-Glucose (^15^N-^13^C labeling). Biomass was grown at 37 °C until OD_600_ ∼ 0.6 and induction of protein expression was achieved using 0.25 mM IPTG at 15 °C during 36 h. Bacteria were harvested (5000 g, 20 min) and pellets resuspended in lysis buffer (50 mM Tris pH 8, 500 mM NaCl, 1 M urea, 2 mM DTT) supplemented with 0.3 mg ml^−1^ lysozyme and EDTA-free protease inhibitor cocktail (Roche) prior to pressure-assisted lysis using a French press system. After three cycles at 1500 bar the lysate was finally centrifuged at 12,500*g* for 30 min and the supernatant was collected. This was loaded on a histidine affinity chromatography column (5 ml HisTrap FF crude, GE) equilibrated with the loading buffer (50 mM Tris pH 8, 500 mM NaCl, 1 M Urea and 2 mM DTT) and a flow rate of 2.5 ml.min^−1^ at 4 °C. The loaded column was washed with 40 mM imidazole (10 CV) and the protein was eluted with 150 mM imidazole (5 CV). The elution product was dialyzed against the Prescission digestion buffer (50 mM Tris-HCl pH 7.4, 150 mM NaCl, 10 mM EDTA, and 1 mM DTT) for 2 h. Digestion was achieved with Prescission protease (1:500 w/w ratio) at 4 °C overnight in order to remove His_6_-MBP tag. The digestion mix was then dialyzed against the loading buffer for histidine affinity chromatography (50 mM Tris pH 8, 500 mM NaCl, 1 M urea, 2 mM DTT). Digested Mcl-1 ΔPESTΔTM was isolated in the flowthrough fraction upon loading the digestion mix onto a histidine affinity chromatography column and washing with the loading buffer (5 CV). The protein was concentrated up to 500 μM and further purified using Superdex 200 10/300 column equilibrated with the storing buffer (20 mM Tris pH 7, 300 mM NaCl, 0.5 mM EDTA, 2 mM DTT). Finally, Mcl-1 ΔPESTΔTM was concentrated up to 500 μM and stored at −80 °C.

### BH3 peptides derived from TCTP and Mule

Three BH3 peptides derived from TCTP and from the E3 ubiquitine ligase Mule (UniProtKB - Q7Z6Z7) were purchased (Proteogenix) (Fig. S2*A*). We designed the peptide TCTP_BH3_ to encompass the BH3-like sequence of TCTP from residue D11 to E32, as defined in (19). We also designed the TCTP_BH3D16I_ peptide containing the single amino-acid mutant D16I in the BH3 region to restore the hydrophobic residue found in canonical BH3 motifs at this position h1. Finally, we designed the Mule_BH3_ peptide to contain the Mule BH3 region and to have the same length as the above-mentioned TCTP-derived peptides. All peptides were dialyzed against milliQ water in 1 kDa MWCO membrane and vacuum-dried before use in NMR studies.

### Nuclear magnetic resonance

NMR measurements were performed using Bruker AVIII HD 950 MHz and AVIII 800 MHz spectrometers equipped with TCI cryoprobes. For each experiment in this study, detailed experimental conditions are given in the caption of the corresponding figures. Experiments were carried out at 35 °C under acidic conditions (pH 6.5) for peptide studies, while experiments involving full-length TCTP and Mcl-1 were performed under alkaline conditions (pH 8) to limit solubility issues upon complex formation and to increase the stability of the complexes.

2D ^1^H-^15^N correlation spectra were collected using the ^15^N SOFAST-HMQC experiment (59). We completed sequence-specific backbone assignment of ^15^N-^13^C labeled proteins using classical 3D triple resonance experiments with BEST-TROSY principle (60, 61, 62) as implemented in NMRlib package (63) and on the basis of available assignment data for TCTP (41, 42) and Mcl-1 (58). The same 3D NMR experiments were used for *de novo* backbone assignment of labeled TCTP and Mcl-1 in complex with partners. 2D and 3D correlation spectra were processed with Topspin 3.5 (Bruker) and analyzed with CCPNMR software 2.4 (64). Combined ^1^H-^15^N chemical shift perturbations (Δδ_comb_) were calculated according to the equation Δδ_comb_ = (Δδ^1^H + 0.14Δδ^15^N)^1/2^, where Δδ^1^H and Δδ^15^N are the chemical shift perturbations (in ppm) for ^1^H and ^15^N resonances, respectively (65). For the determination of the dissociation constant (K_D_) of the complex of TCTP BH3-like peptide, we used the TITAN line shape analysis software (v1.6) (38) to analyze a titration series of 11 2D ^15^N SOFAST-HMQC experiments. Given a fixed ^15^N labeled Mcl-1 concentration (100 μM), the peptide titration points were as follows (μM): 0, 10, 20, 30, 40, 50, 100, 150, 200, 300, and 500. We used a two-state binding model, and we defined regions of interest for the following Mcl-1 residues in the TITAN interface: S183, T191, E225, T226, A227, V299, and T301. For all selected residues, the corresponding cross-peaks were isolated and exhibited significant chemical shift perturbations along the titration.

### SEC-SAXS experiments

SEC-SAXS experiments were performed on the SWING beamline at the SOLEIL synchrotron (Saint-Aubin, France). All experiments were done at 37 °C in the following buffer: 50 mM CHES pH 9, 50 mM NaCl, and 2 mM TCEP (Table S1). Isolated TCTP and Mcl-1 were prepared at a final concentration of 500 μM. The TCTP−Mcl-1 complex was prepared as an equimolar mix of the two proteins (500 μM each) and incubated for 2 h. For each sample, a volume of 75 μl was injected onto a size exclusion column (Superdex 200-10/300, GE Healthcare) and eluted directly into the SAXS flow-through capillary cell with a flow rate of 0.5 ml.min^−1^. The SAXS data were recorded using an EigerX 4M detector at a distance of 2 m with a definition of the momentum transfer *q*: *q* = *4πsin(θ)/λ* with 2*θ* as the scattering angle and *λ* the X-ray wavelength (1.033 Å for these experiments). The overall SEC-SAXS setup has already been described previously (66, 67, 68).

In total, 900 SAXS frames were collected continuously upon elution with a frame duration of 1.99 s and a dead time between frames of 0.01 s 180 frames accounting for buffer scattering were collected before the void volume. Data reduction to absolute units, buffer subtraction, and averaging of identical frames corresponding to the elution peak was performed with the SWING in-house software FOXTROT (66) and US-SOMO (69). US-SOMO was also used to estimate the MW based on the volume of correlation (70). Data analysis yielding the intensity at *q* = 0 (*I*(0)), radius of gyration (Rg), and maximal diameter of the protein (Dmax) were conducted with the PRIMUS software from the ATSAS Suite (71). Dammif *via* Primus (72) was also used to compute the molecular envelope of free Mcl-1 and in complex with TCTP.

### Circular dichroism spectroscopy

The far-UV (180–260 nm) Circular Dichroism (CD) experiments were recorded at 25 °C under N_2_ atmosphere using a JASCO J-810 spectropolarimeter to observe the secondary structure content of proteins (73). The CD cell path length was 0.1 mm. Ten accumulations at 100 nm.min^−1^ with data pitch 0.2 nm and digital integration time of 1 s were recorded for each sample. Detector voltage never exceeded 700 V. For the simulation of CD spectra, the BeStSel online software (http://bestsel.elte.hu) was used (74). For each CD experiment in this study, the detail of buffer conditions is given in the caption of the corresponding figures.

### Protein-peptide docking with HADDOCK

We performed molecular docking using HADDOCK 2.2 algorithm (75, 76) to inform on the possible interfaces between Mcl-1 protein and BH3 peptides derived from TCTP. HADDOCK can integrate experimental data such as chemical shift perturbation to predict an accurate model of a protein complex. Therefore, “active” residues were defined by comparing ^15^N SOFAST-HMQC spectra of ^15^N-labeled Mcl-1 in complex with Mule_BH3_ or TCTP_BH3D16I_. Since the sequences of Mule_BH3_ and TCTP_BH3D16I_ are different, it is expected that spectral differences inform on Mcl-1 residues in direct contact with the BH3 peptide ligand (Fig. S3). Correspondingly, the list of Mcl-1 “active” residues provided to HADDOCK interface included: Q221, H252, F254, S255, T259, W261, G262, R263, I264, V265, and T266. For BH3 peptides, the list of “active” residues included the positions h1, h2, h3, and h4 (Fig. S2*A*). For Mcl-1 and BH3 peptides, the definition of “passive” residues was set to “automatic”. HADDOCK requires a target and ligand as inputs, herein structural models of Mcl-1 and the BH3 peptide. For docking simulation, we used the bound Mcl-1 conformation extracted from the crystal structure of Mcl-1 in complex with Mule BH3 motif (T. Song, unpublished results) (PDB code: 5C6H). For TCTP_BH3_ and TCTP_BH3D16I_ peptides, the α-helical Mule_BH3_ structure was used as a template for further *in silico* mutagenesis in PyMol (77). Of note, we used an α-helical model for TCTP_BH3_ because it is the conformation found in the crystallographic structure of TCTP_BH3_ in complex with the Mcl-1 homolog Bcl-xL (19).

### Limited proteolysis

Full-length TCTP and its complex with Mcl-1 were analyzed by limited proteolysis in order to evaluate to which extent the proteins remain packed and protected from proteolysis upon complex formation. Experiments were carried out on concentrated samples (500 μM each protein) considering both isolated TCTP or Mcl-1 proteins as well as an equimolar mix of the two. Limited proteolysis was achieved at a low (1 μg ml^−1^) concentration of chymotrypsin or trypsin proteases or with an intermediate (5 μg ml^−1^) protease concentration. As a control of the efficiency of the proteases and to obtain the full degradation pattern, complete digestion was achieved at a high (100 μg ml^−1^) protease concentration. Samples were prepared in 50 mM EPPS pH 8, 50 mM NaCl, 2 mM TCEP and incubated for 6 h at 37 °C prior to heat-denaturation and SDS-PAGE analysis.

## Data availability

A CcpNmr (64) session with ^1^H-^15^N SOFAST-HMQC spectra of isolated ^15^N-Mcl-1 ΔPESTΔTM (Mcl-1) and in complex with TCTP BH3-like peptide (TCTPBH3) or TCTP BH3-like peptide D16I mutant (TCTPBH3D16I) or the E3 ubiquitine ligase Mule BH3 peptide (MuleBH3) are available here: https://github.com/Synthaze/JBC-FileS1/tree/main/FileS1/Mcl1_BH3s. The corresponding backbone assignments are provided within the CcpNmr session. Experiments were recorded at 950 MHz and 308 K in the following buffer: 50 mM MES pH 6.5, 50 mM EPPS, 50 mM NaCl, 2 mM TCEP in 5% D_2_O / 95% H_2_O.

## Supporting information

This article contains supporting information (64, 78).

## Conflict of interest

The authors declare that they have no conflicts of interest with the contents of this article.
