# Supplementary material for: Structural transitions in TCTP tumor protein upon binding to the anti-apoptotic protein family member Mcl-1
Source: J Biol Chem. 2023 May 16;299(7):104830. doi: 10.1016/j.jbc.2023.104830 (PMC10333598; doi:10.1016/j.jbc.2023.104830)
Supplement: Supporting information [file mmc1.pdf]

Supporting Information for:

Structural transitions in TCTP tumor protein upon binding to the anti-apoptotic protein family member Mcl-1

Supplementary figures

```
TCTP Full Length (TCTP FL)
-1  1
 GP MIIYRDLISH DEMFSDIYKI REIADGLCLE VEGKMVSRTE GNIDDSLIGG NASAEGPEGE GTESTVITGV

    DIVMNHHLQE TSFTKEAYKK YIKDYMKSIK GKLEEQRPER VKPFMTGAAE QIKHILANFK NYQFFIGENM
                                      172
    NPDGMVALLD YREDGVTPYM IFFKDGLEME KC

Mcl-1 ΔPESTΔTM
-1  1 (172)
 GP DELYRQSLEI ISRYLREQAT GAKDTKPMGR SGATSRKALE TLRRVGDGVQ RNHETAFQGM LRKLDIKNED

    DVKSLSRVMI HVFSDGVTNW GRIVTLISFG AFVAKHLKTI NQESCIEPLA ESITDVLVRT KRDWLVKQRG
                     156 (327)
    WDGFVEFFHV EDLEGG
```

Figure S1: **Primary sequence of FL-TCTP and Mcl-1 ΔPEST ΔTM used for interaction studies.** For TCTP Full Length (FL-TCTP), the N-Terminal GP residues (grey) originate from the TEV cleavage site. Residues M1 to C172 corresponds to the full length sequence of human TCTP protein (UniProtKB P13693). For Mcl-1 ΔPEST ΔTM, the N-Terminal GP residues originate from the Prescission cleavage site. Residues D1 to G156 corresponds to the D172 to G327 segment of human Mcl-1 (UniProtKB Q07820).

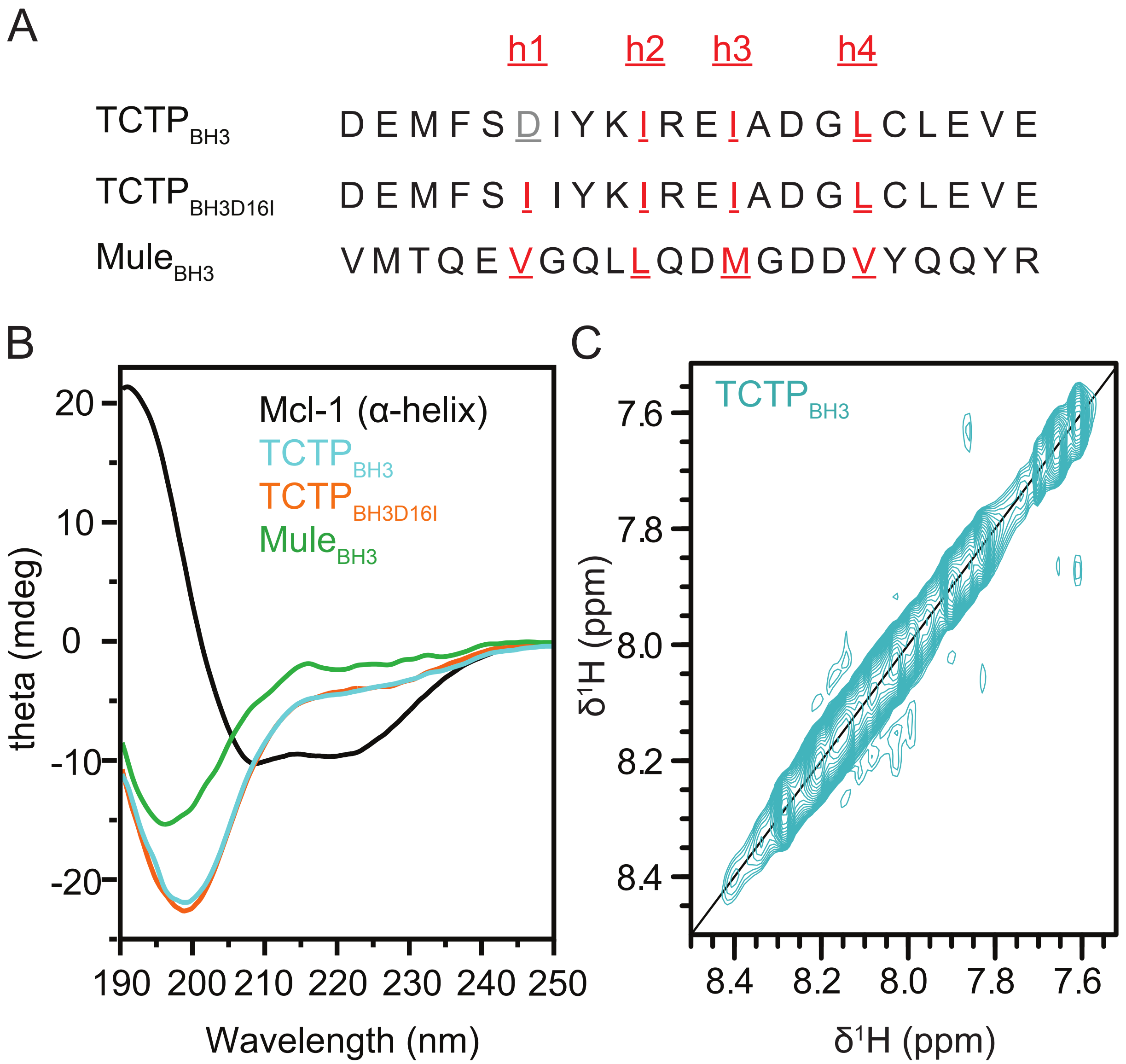

Figure S2: **Primary sequences and structural characterization of BH3 peptides related to TCTP and Mule.** (A) Primary sequence of the TCTP BH3-like peptide ($TCTP_{BH3}$), the canonical D16I mutant ($TCTP_{BH3D16I}$) and the canonical BH3 peptide from the E3 ubiquitine ligase Mule ($Mule_{BH3}$). Conserved position h1, h2, h3, h4 in canonical BH3 motifs are highlighted (red). $TCTP_{BH3}$ and $Mule_{BH3}$ peptide constructs correspond to the segment 11-32 and 1971-1992 in the full-length TCTP and Mule, respectively. (B) Far-UV (190-250 nm) CD experiments with $TCTP_{BH3}$ (100 µM, cyan), $TCTP_{BH3D16I}$ (100 µM, orange), $Mule_{BH3}$ (100 µM, green) and an α-helical reference Mcl-1 ΔPEST ΔTM (Mcl-1) (100 µM, black). Experiments were carried out at 298 K in 2.5 mM phosphate buffer pH 6.5. (C) ^{1}H-^{1}H NOESY spectrum (τ_m = 200 ms) of $TCTP_{BH3}$. Experiment was recorded at 800 MHz and 278 K in the following buffer: 50 mM MES pH 6.5, 50 mM NaCl, 2 mM TCEP in 5 % D_2O / 95 % H_2O.

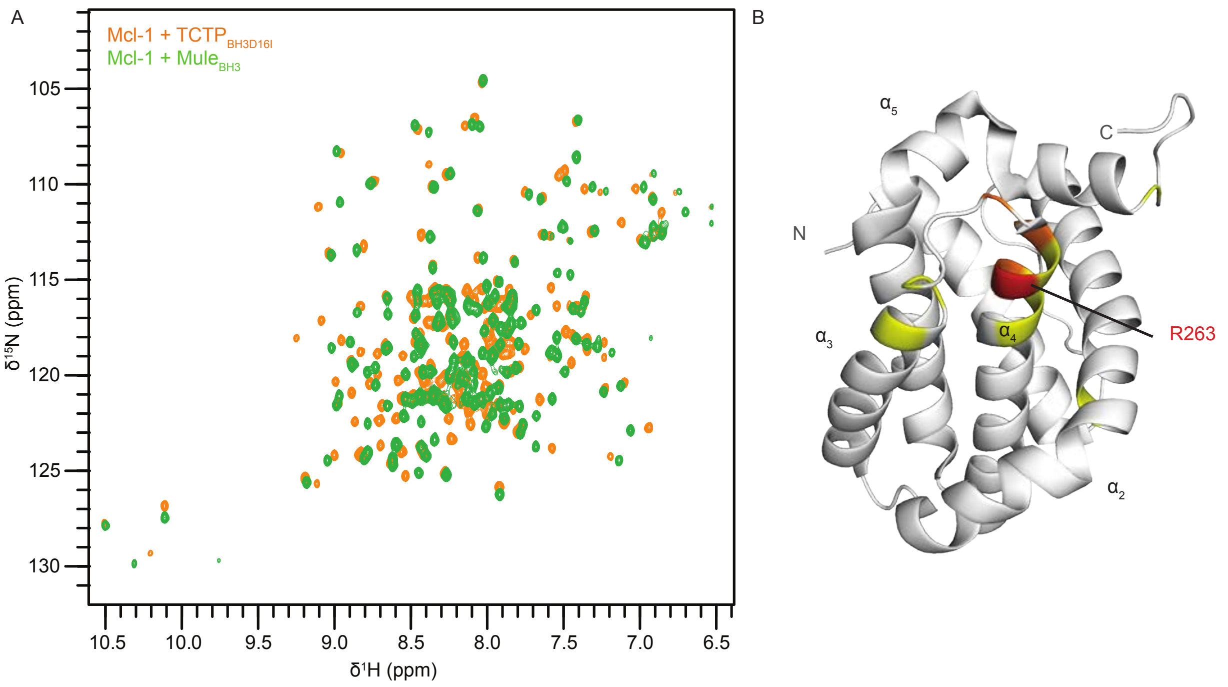

Figure S3: **Comparison of $\mathrm{TCTP_{BH3D16I}}$ and $\mathrm{Mule_{BH3}}$ binding to Mcl-1.** (A) Overlay of ^{15}N SOFAST HMQC spectra from ^{15}N-Mcl-1 ΔPEST ΔTM (Mcl-1) (100 µM) in complex with TCTP BH3-like peptide D16I mutant ($\mathrm{TCTP_{BH3D16I}}$) (orange) or $\mathrm{Mule_{BH3}}$ (green). (B) Mapping of combined ^{1}H-^{15}N chemical shift perturbations between ^{15}N-Mcl-1 ΔPEST ΔTM (Mcl-1) (100 µM) in complex with TCTP BH3-like peptide D16I mutant ($\mathrm{TCTP_{BH3D16I}}$) or $\mathrm{Mule_{BH3}}$ ($\Delta\delta_{comb} > 0.9$, red; $0.9 > \Delta\delta_{comb} > 0.65$, orange; $0.65 > \Delta\delta_{comb} > 0.4$, yellow; $0.4 > \Delta\delta_{comb}$, white) on the NMR structure of Mcl-1. Experiments were recorded at 950 MHz and 308 K in the following buffer: 50 mM EPPS pH 8, 50 mM NaCl, 2 mM TCEP in 5 % D_2O / 95 % H_2O.

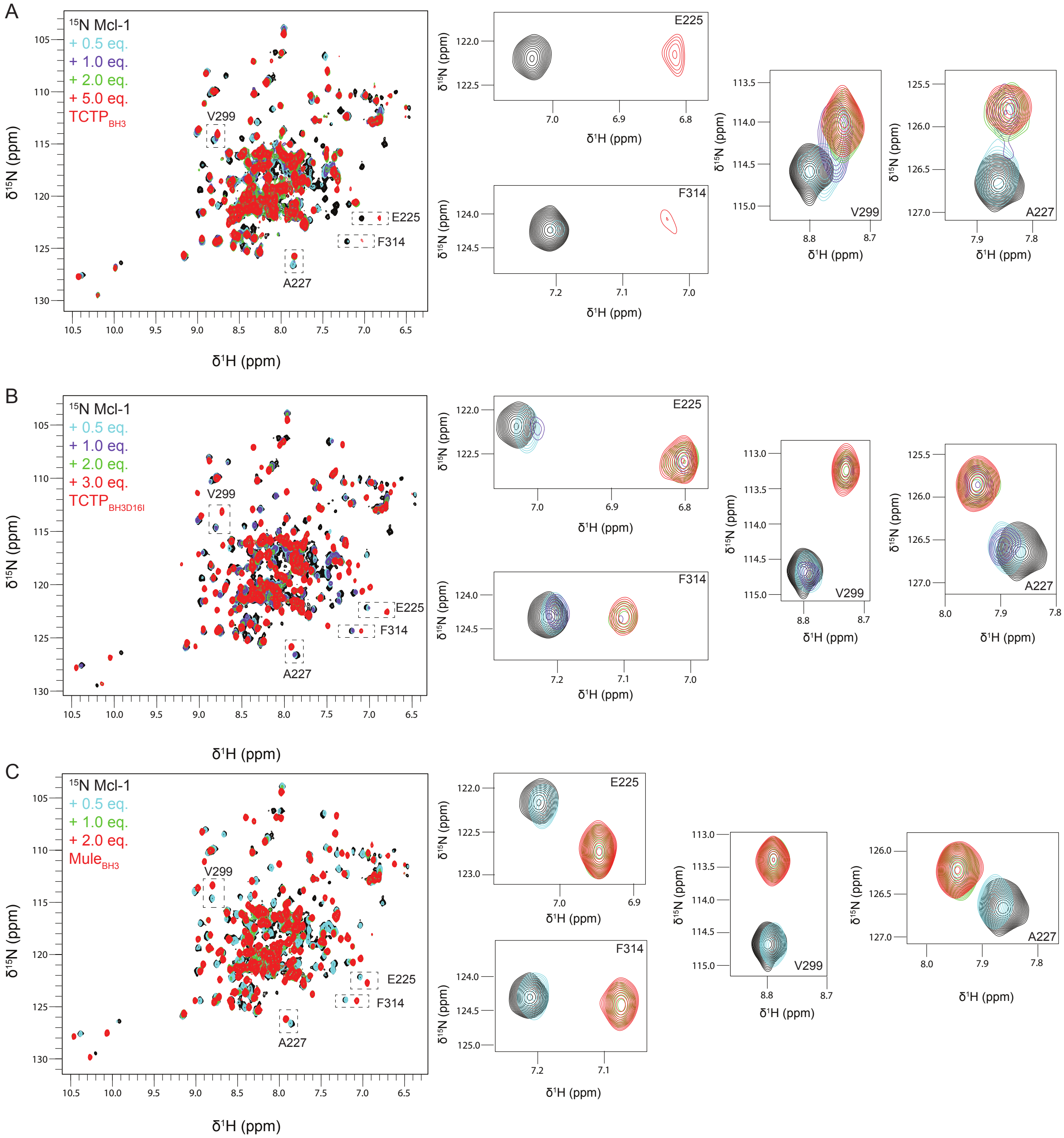

Figure S4: **NMR titrations of Mcl-1 with BH3-derived peptides and exchange regimes.** Overlay of ^{15}N SOFAST HMQC spectra from ^{15}N-Mcl-1 ΔPEST ΔTM (Mcl-1) (100 µM) and upon addition of increasing amount of (A) TCTP BH3-like peptide (TCTP$_{BH3}$) or (B) TCTP BH3-like peptide D16I mutant (TCTP$_{BH3D16I}$) or (C) Mule BH3 peptide (Mule$_{BH3}$). A close-up view for NMR crosspeaks corresponding to residues E225, F314, V299 and A227 is given to illustrate the exchange regime along the titration. Experiments were recorded at 950 MHz and 298 K in the following buffer: 50 mM MES pH 6.5, 50 mM NaCl, 2 mM TCEP in 5 % D_2O / 95 % H_2O.

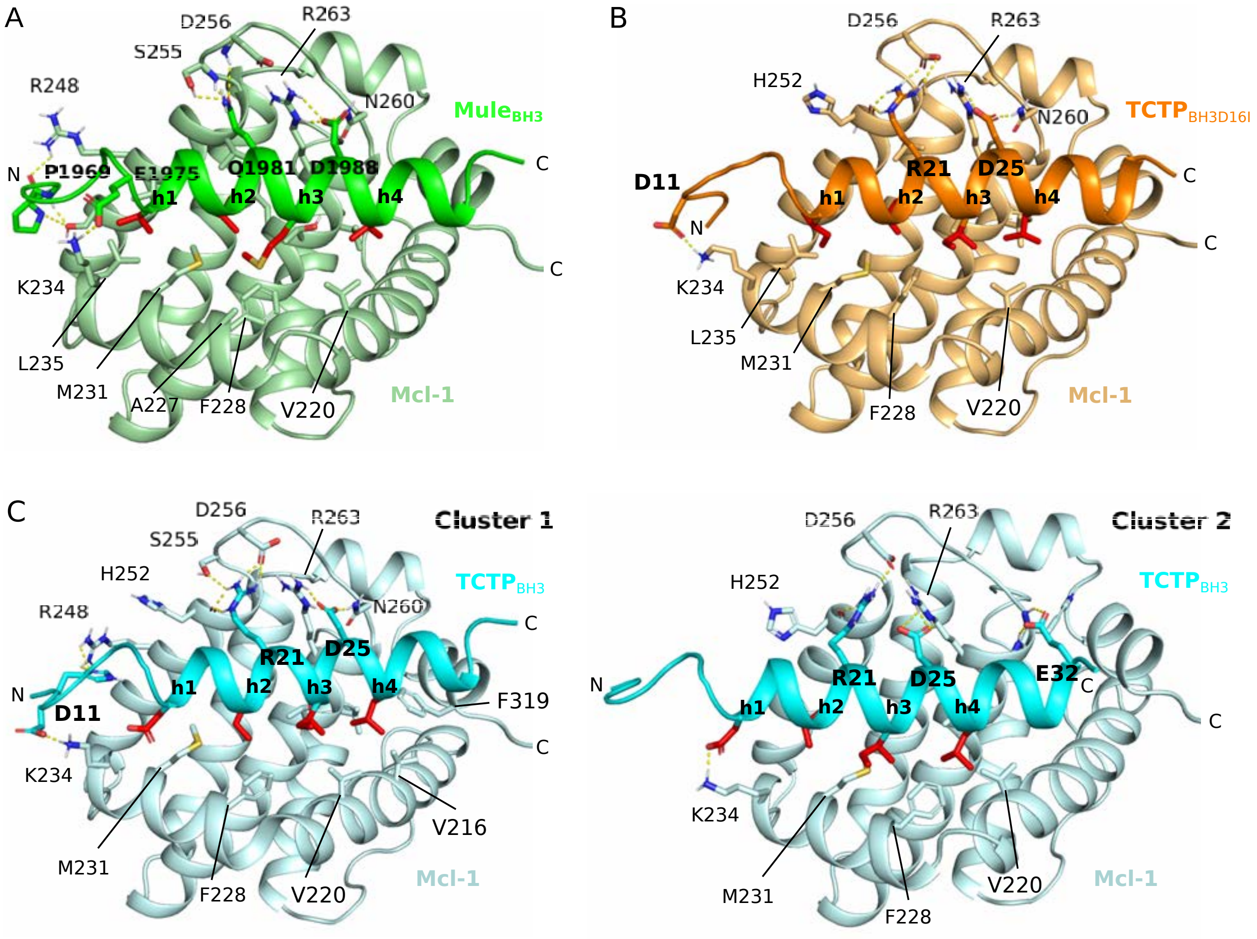

Figure S5: **Interaction interfaces between Mcl-1 and BH3 peptides in HADDOCK docking models.** (A) Representative structure of the top ranked cluster for Mcl-1/Mule$_{\mathrm{BH3}}$. (B) Representative structure of the top ranked cluster for Mcl-1/TCTP$_{\mathrm{BH3D16I}}$. (C) Representative structure of (left) cluster 1 and (right) cluster 2 for Mcl-1/TCTP$_{\mathrm{BH3}}$. In (A), (B) and (C), bold and regular labels indicate BH3 and Mcl-1 residues, respectively. In BH3 peptides, residues in h1-4 positions (red sticks) and surrounding hydrophobic residues in Mcl-1 (sticks) are highlighted. Electrostatic contacts (yellow dashed lines) and contributing residues (sticks) are highlighted.

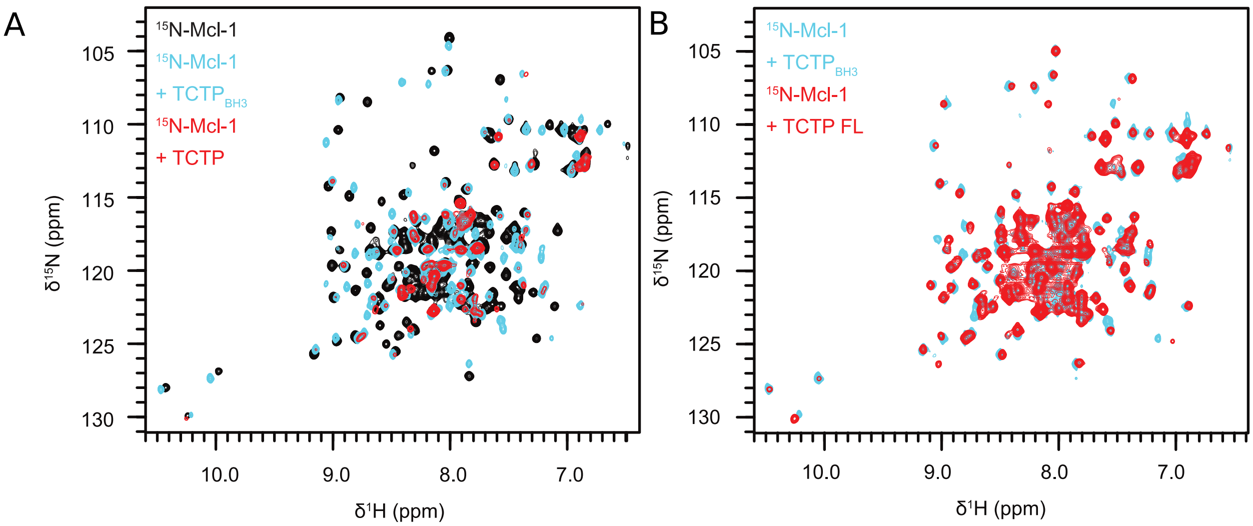

Figure S6: **Comparison of TCTP and TCTP$_{BH3}$ binding to Mcl-1.** (A) Overlay of ^{15}N SOFAST HMQC spectra from isolated ^{15}N-Mcl-1 (100 µM, black) and in complex with TCTP BH3-like peptide (TCTP$_{BH3}$) (cyan) or full length TCTP (red). (B) Overlay of ^{15}N SOFAST HMQC spectra from ^{15}N-Mcl-1 ΔPEST ΔTM (Mcl-1) (100 µM) in complex with TCTP BH3-like peptide (TCTP$_{BH3}$) (cyan) or full length TCTP (red). Spectral intensity was scaled for comparison of chemical shift profiles in both conditions. Experiments were recorded at 950 MHz and 308 K in the following buffer: 50 mM EPPS pH 8.0, 50 mM NaCl, 2 mM TCEP in 5 % D_2O / 95 % H_2O.

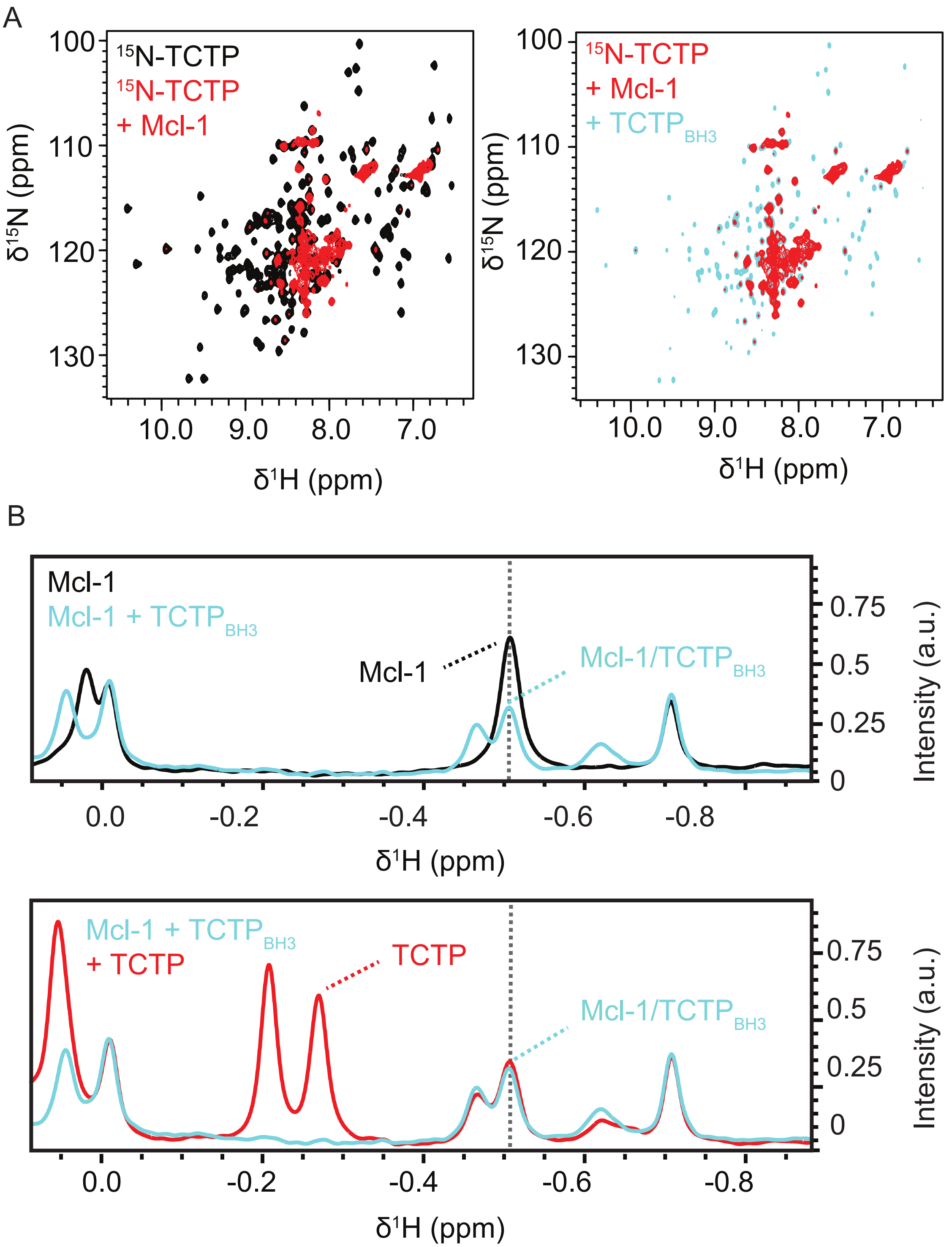

Figure S7: **Relative binding capacity of TCTP$_{\mathbf{BH3}}$ and FL-TCTP to Mcl-1.** (A) Overlay of ^{1}H spectra from isolated Mcl-1 ΔPEST ΔTM (Mcl-1) (100 µM, black), upon addition of TCTP$_{BH3}$ (4 eq., blue), FL-TCTP (2 eq., red), and after incubation time (2 hrs) (green). (B) Overlay of ^{15}N SOFAST HMQC spectra from isolated ^{15}N-TCTP (100 µM, black), in complex with unlabeled Mcl-1 ΔPEST ΔTM (Mcl-1) (2 eq.) (red) and upon addition of TCTP$_{BH3}$ (2 eq.) (cyan). Experiments were recorded at 950 MHz and 308 K in the following buffer: 50 mM EPPS pH 8, 50 mM NaCl, 2 mM TCEP in 5 % D_2O / 95 % H_2O.

Supplementary tables

	Mcl-1	TCTP/Mcl-1
Organism	Human	Human
Source	E. Coli	E. Coli
Description - sequence (including tags) + bound ligands/modifications, etc.	See Supp. Fig. S1	See Supp. Fig. S1
Extinction coefficient	19480 $M^{-1}.cm^{-1}$	19480 + 11920 = 31400 $M^{-1}.cm^{-1}$
M from chemical composition	17765.21 Da	17765.21 + 19652.39 = 37417.6 Da
For SEC-SAS, loading volume/concentration, flow rate	75µL/500µM, 0.5mL/min	75µL/500µM, 0.5mL/min
Solvent details	50 mM CHES pH 9, 50 mM NaCl and 2 mM TCEP	50 mM CHES pH 9, 50 mM NaCl and 2 mM TCEP
Guinier Analysis	Mcl-1	TCTP/Mcl-1
I(0)	0.0078 +/- 0.00001	0.0036 +/- 0.00001
R_g	1.758 +/- 0.004 nm	2.102 +/- 0.008 nm
qR_g range	0.14-1.3	0.22-1.29
M from *I*(0) (ratio to expected value)	21.7 kDa (1.22)	34.7 kDa (0.93)
P(*r*) analysis	Mcl-1	TCTP/Mcl-1
V_c	206	283

Table S1: **Reporting of essential Small Angle Scattering (SAS) data acquisition, sample details, data analysis, modelling fitting and software used.** We used a reporting template from [78] to provide relevant details of experiments performed with biomolecules in solution at SOLEIL synchrotron (Saint-Aubin, France).

References

[64] Vranken, W. F., Boucher, W., Stevens, T. J., Fogh, R. H., Pajon, A., Llinas, M., et al. (2005). The CCPN data model for NMR spectroscopy: development of a software pipeline. *Proteins*, **59**, 687–696.

[**78**] Trewhella, J., Duff, A. P., Durand, D., Gabel, F., Guss, J. M., Hendrickson, W. A., et al. (2017). 2017 publication guidelines for structural modelling of small-angle scattering data from biomolecules in solution: an update. *Acta Crystallogr.*, **73**, 710–728.
